# Functional insights into *Streptomyces* isolates containing both clavulanic acid-like and carbapenem biosynthetic gene clusters

**DOI:** 10.1128/msphere.00188-25

**Published:** 2025-08-25

**Authors:** Kapil Tahlan, Arshad Ali Shaikh, Jingyu Liu, Kajal Gupta, Nader AbuSara, Santosh Kumar Srivastava, Adau Deng, Ayla Rouah, Madelyn Joan Swackhamer

**Affiliations:** 1 Department of Biology, Memorial University of Newfoundland 120908, St. John's, Newfoundland and Labrador, Canada; University of Nebraska Medical Center College of Medicine, Omaha, Nebraska, USA

**Keywords:** *Streptomyces*, β-lactam, clavulanic acid, carbapenem, β-lactamase inhibition, biosynthetic gene cluster

## Abstract

β-Lactam antibiotics and β-lactamase inhibitor combinations are essential for combating antimicrobial resistance, with many β-lactams, including clavulanic acid (CA), themselves being products of specialized metabolic pathways in bacteria. CA is a potent β-lactamase inhibitor, and in known producers such as *Streptomyces clavuligerus*, it is co-produced with the β-lactam antibiotic cephamycin C, and their biosynthetic gene clusters (BGCs) are always located adjacent on the chromosome. However, CA-like BGCs have also been identified in other bacteria, often without an accompanying cephamycin C BGC. Similarly, carbapenem BGCs (a subclass of β-lactams), such as those responsible for producing MM 4550, a member of the olivanic acid complex with both antibiotic and β-lactamase inhibitory properties, are also found in *Streptomyces* species. This study investigated antimicrobial and β-lactamase inhibitory activity production in *Streptomyces pratensis* and 10 environmental *Streptomyces* isolates (JAC strains) containing CA-like and MM 4550-like BGCs but lacking cephamycin C BGCs. While the examined isolates do not produce CA, they synthesize predicted monocyclic β-lactam precursors of CA, which potentially represent a previously unrecognized, primordial form of β-lactamase inhibitor. Several JAC isolates also exhibited both β-lactamase inhibitory and β-lactam antibiotic activities, indicating that the carbapenem BGC is active in these strains. Gene disruption analysis confirmed that MM 4550-like carbapenem BGCs contribute to both antimicrobial and β-lactamase inhibitory activities, whereas CA-like clavam BGCs only contribute to β-lactamase inhibition. The findings also suggest that both β-lactam BGC types co-occur in nature more frequently than previously recognized, possibly with functional significance and potential applications in the discovery of novel antibiotic-inhibitor combinations.

**IMPORTANCE:**

The global rise of antimicrobial resistance calls for innovative strategies to preserve the efficacy of existing antibiotics and identify new therapeutic agents. This study explores naturally occurring β-lactamase inhibitors and antibiotics beyond well-characterized systems. Investigation of clavulanic acid (CA)-like and MM 4550-like biosynthetic gene clusters (BGCs) in *Streptomyces pratensis* and related environmental isolates revealed a broader occurrence of monocyclic β-lactam precursors and dual-function carbapenems in nature. These findings offer new insights into β-lactam co-production and further indicate that unlinked β-lactam BGCs may have functional significance. The study also highlights the importance of exploring silent counterparts of known BGCs as potential sources of bioactive metabolites, enhancing our understanding of β-lactam BGC diversity and evolution. Notably, it identifies β-lactamase inhibitor and antibiotic-producing strains, opening new avenues for discovering antibiotic-inhibitor combinations of relevance.

## INTRODUCTION

**Fig 1:**
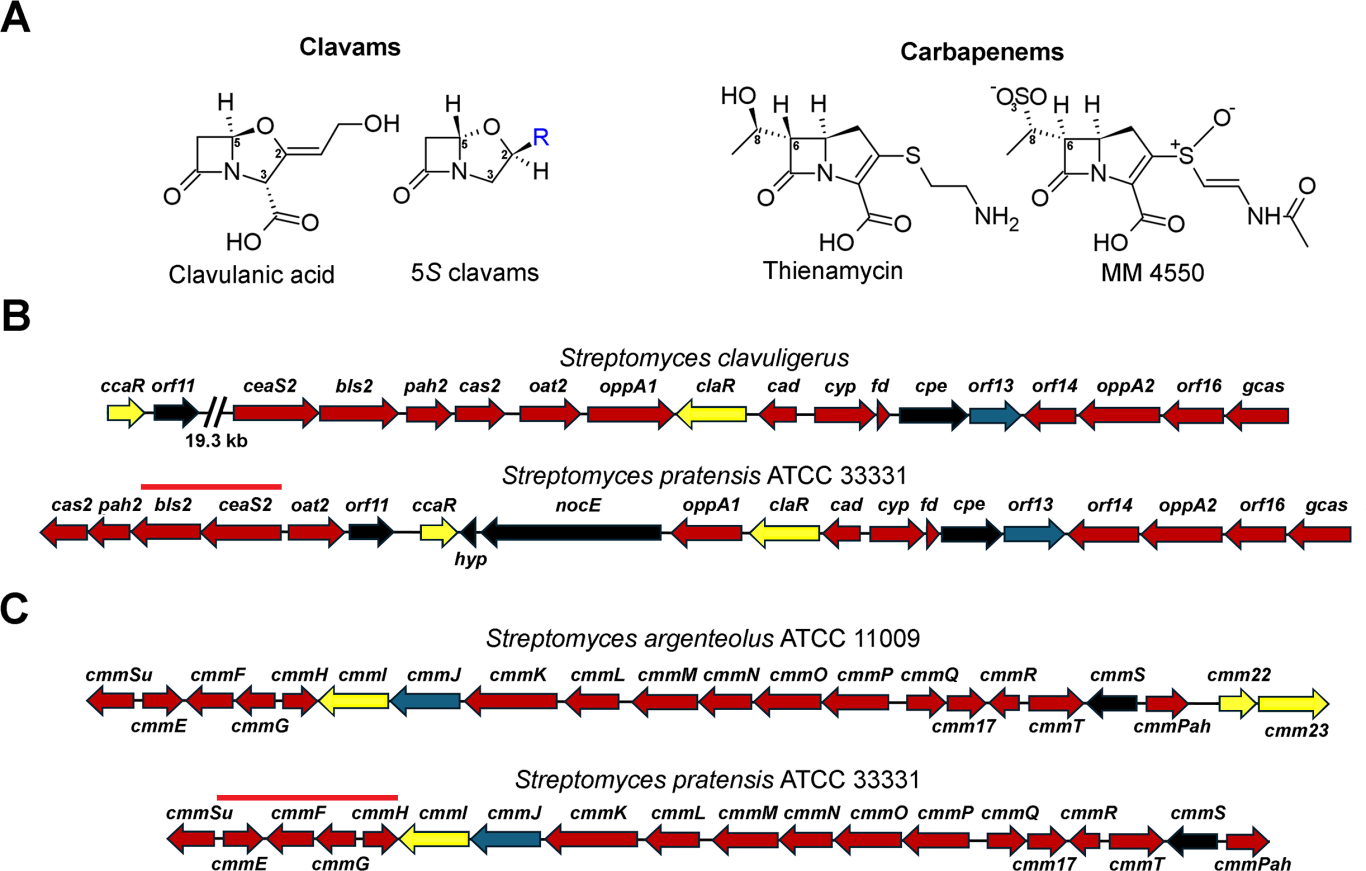
Clavams, carbapenems, and their biosynthetic gene clusters (BGCs) in select *Streptomyces* species. (**A**) The structures of CA and the 5*S* clavams (where R represents different side chain substituents) are shown as representative compounds. Additionally, the structures of select carbapenems whose biosynthesis has been characterized in different *Streptomyces* species are included. Ring atoms responsible for stereochemical and other differences are numbered for reference. (**B**) Comparison of the CA BGC in *Streptomyces clavuligerus* with the CA-like gene cluster in *Streptomyces pratensis*. Certain genes upstream of *ceaS2*, which belong to the cephamycin C gene cluster in *S. clavuligerus,* are also included for reference since their homologs are also present in the CA-like BGC from *S. pratensis*. (**C**) Relative organization of the MM 4550 BGC in *Streptomyces argenteolus* and the similar gene cluster in *S. pratensis*. (**B and C**) The arrows indicate genes, with the arrowheads showing the direction of transcription. The arrows are color-coded based on function: red indicates biosynthetic genes, blue indicates transport, yellow indicates regulatory, and black represents resistance genes or those with unclear function within the respective BGCs. The genes from the CA-like and MM 4550-like BGCs of *S. pratensis* that were targeted for mutant construction are indicated by solid red lines in the figure.

The clavams and carbapenems represent two important sub-classes of β-lactam compounds with significant roles in human medicine (1). Unlike other β-lactams such as penicillins, cephalosporins, and monobactams, the clavams and carbapenems are produced by unrelated biosynthetic pathways that do not involve non-ribosomal peptide synthetases (2). Clavulanic acid (CA, Fig. 1A) is a clavam with 5*R* stereochemistry and is an inhibitor of many class A and some class D serine β-lactamases (3), enzymes that inactivate β-lactam antibiotics, thereby conferring resistance. Consequently, CA is clinically used in combination with some β-lactam antibiotics to treat infections caused by β-lactamase-producing organisms (1). The pharmaceutical industry produces CA exclusively through the fermentation of *Streptomyces clavuligerus*, as large-scale total synthesis is not commercially feasible. In addition, only two other bacterial species, *Streptomyces jumonjinensis* and *Streptomyces katsurahamanus*, are known to synthesize CA (4).

The ability to produce other clavam metabolites with 5*S* stereochemistry (Fig. 1A), which lack β-lactamase inhibitory activity, appears to be more widespread in nature than CA biosynthesis. The biosynthetic gene clusters (BGCs) responsible for producing some of the 5*S* clavams have also been identified (4). In comparison, the genes involved in the biosynthesis of CA are always clustered with those required for producing the β-lactam antibiotic cephamycin C, and these two metabolites are typically co-produced in nature (5, 6). Even though CA has 5*R* stereochemistry, the biosynthetic pathway leading to its formation includes monocyclic β-lactam intermediates and 5*S* clavams (7). The 5*S* to 5*R* stereochemical inversion and the resulting gain of potent β-lactamase inhibitory activity occur at a later stage in the CA biosynthetic pathway. In addition, CA and its biosynthetic precursors do not exhibit significant antibiotic activity; however, it is not known if early intermediates possess some other properties. Gene clusters resembling the CA BGC are also found in other bacteria (6, 8), including *Streptomyces pratensis* ATCC 33331 (Fig. 1B) (4). However, in these cases, the clusters are not adjacent to the cephamycin C BGC (6), and the production of CA has not been demonstrated to occur in such organisms (9). Therefore, these singleton CA-like gene clusters in CA non-producing organisms are either silent or could be involved in the biosynthesis of other metabolites.

In addition to the CA-like BGC, *S. pratensis* also harbors a cryptic carbapenem BGC (10). To date, more than 50 naturally occurring carbapenems have been reported, and BGCs from different *Streptomyces* involved in the biosynthesis of thienamycin (11) and MM 4550 (12) have been characterized. Thienamycin is one of the most potent and broad-spectrum natural antibiotics known and the first complex carbapenem to be described, whereas MM 4550 belongs to the olivanic acid complex (13), a group of carbapenems that exhibit both β-lactamase inhibitory and antibiotic activities (Fig. 1A). MM 4550 differs from thienamycin in its stereochemistry and is sulfonated at the C8 position (12), which is characteristic of the olivanic acids. Therefore, the dual activities of certain carbapenems make them attractive candidates for further studies. However, issues related to stability and target penetration have hindered the clinical progress of the olivanic acids (14).

The coordinated production of β-lactams from different subclasses with varying activities, such as cephamycin C (a β-lactam antibiotic) and CA (a β-lactamase inhibitor), is well established (15). In *Streptomyces cattleya*, the biosynthesis of the β-lactam antibiotics thienamycin and cephamycin C is also coordinately regulated (16). The presence of two unrelated but functionally complementary β-lactam BGCs in *S. pratensis*, which are not physically linked, raises many questions. The co-production of clavams and carbapenems has not been documented previously and could provide valuable insights into the occurrence and functions of such BGCs in natural environments. In this study, we aimed to examine the activities associated with the CA-like and carbapenem gene clusters in *S. pratensis* and other environmental organisms containing similar BGCs to test this hypothesis.

## RESULTS AND DISCUSSION

*Streptomyces pratensis* ATCC 33331 (henceforth referred to as *S. pratensis*) contains BGCs resembling those responsible for producing CA (4) and carbapenems (Fig. 1A) (10). All genes necessary for CA biosynthesis are present in *S. pratensis* (Fig. 1B), but their arrangement differs from CA BGCs of producers such as *S. clavuligerus* (4). Additionally, the *S. pratensis* CA-like BGC contains homologues of genes typically associated with other β-lactams, such as *orf11* and *ccaR* from the cephamycin C cluster of *S. clavuligerus* (9), as well as *nocE* from the nocardicin A cluster from *Nocardia uniformis* (6, 17). A closer examination of the *S. pratensis* carbapenem BGC revealed that it is almost identical to the MM 4550 BGC from *Streptomyces argenteolus* (Fig. 1C), with similar gene content and organization, except for a missing two-component regulatory system (*cmm22* and *cmm23*) (12). However, CA or MM 4550 has not been detected under standard laboratory culture conditions in *S. pratensis,* and the species has not been reported to produce any β-lactam-associated bioactivities endogenously (9). Therefore, we set out to investigate if the production of metabolites associated with the two β-lactam BGCs in *S. pratensis* could be elicited by altering growth conditions.

### β-lactamase inhibitor production by *S. pratensis*

To investigate the ability of *S. pratensis* to produce β-lactams, we employed the One Strain Many Compounds approach by culturing the bacterium in various types of liquid broths and solid media (Table S1). The production of β-lactamase inhibitory activity was determined using *Klebsiella pneumoniae* ATCC 15380 (Table S2) as an indicator in the presence of penicillin G, a method routinely used for detecting CA in cultures from *S. clavuligerus* (18). Additionally, *Escherichia coli* ESS (a β-lactam-sensitive strain) was used to detect the presence of β-lactam antibiotics, including carbapenems (12).

**Fig 2:**
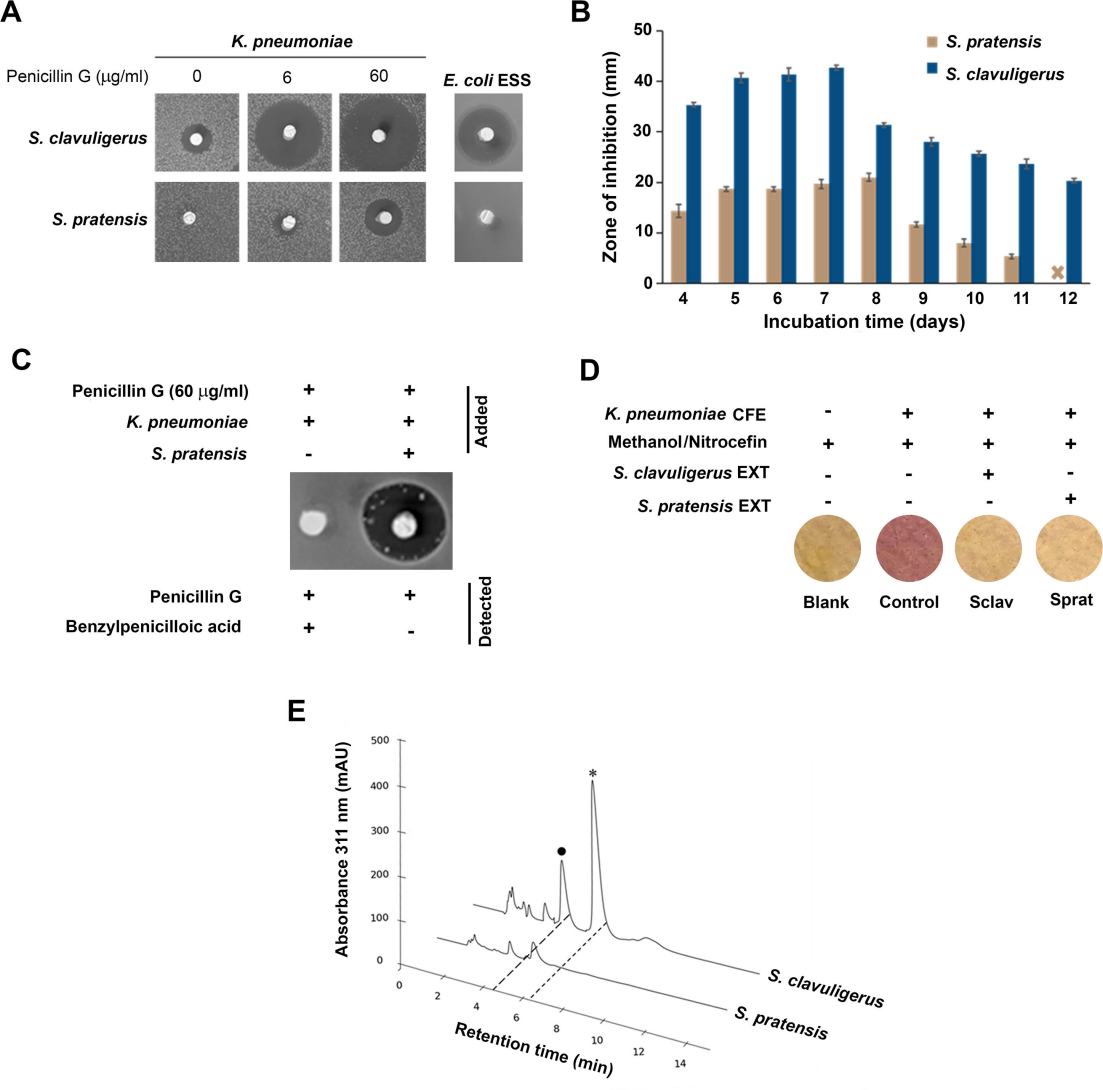
Production of a β-lactamase inhibitor by *S. pratensis*. (**A**) Bioassays using *Klebsiella pneumoniae* as an indicator in the absence and presence of penicillin G (6 and 60 µg/mL). Agar plugs from soy agar cultures of *S. pratensis* and *S. clavuligerus* were tested after 7 and 9 days of growth, respectively. The formation of zones of inhibition in the presence of penicillin G indicates the production of β-lactamase inhibitory activity. Please note that any overlaps or similarities in the images can be attributed to the assay being conducted on a single bioassay plate. (**B**) Time-course analysis of β-lactamase inhibitory activity production in soy agar cultures of *S. pratensis* and *S. clavuligerus* using *K. pneumoniae* in the presence of 60 µg/mL penicillin G. (**C**) Examination of penicillin G hydrolysis by *K. pneumoniae* in the absence and presence of *S. pratensis* soy agar culture plugs. The region of the *K. pneumoniae* culture directly under the plugs was extracted with methanol and subjected to metabolomic analysis. Hydrolysis of penicillin G by the *K. pneumoniae* β-lactamase in the absence of *S. pratensis* plugs is indicated by the production and detection of benzylpenicillinoic acid. (**D**) Demonstration of β-lactamase inhibition using *K. pneumoniae* cell-free extracts (CFE) with nitrocefin as a substrate. Methanol extracts from soy agar cultures of *S. pratensis* were tested, and those from *S. clavuligerus were* included for comparison. Hydrolysis of nitrocefin by β-lactamases leads to the production of a characteristic red color, which was inhibited in reactions containing extracts from the two *Streptomyces* species tested. (**E**) High-performance liquid chromatography (HPLC) analysis of imidazole-derivatized aqueous extracts of soy agar cultures of *S. pratensis* and *S. clavuligerus* using the bondolone method as described in the Materials and Methods. The peak corresponding to clavulanic acid in *S. clavuligerus* extracts is marked with a star, which is absent in the corresponding extracts from *S. pratensis*. The other peak in *S. clavuligerus*, marked with a solid circle, corresponds to 2-hydroxymethyl clavam, a metabolite that does not exhibit β-lactamase inhibitory activity.

It was found that *S. pratensis* only produced β-lactamase inhibitory activity when cultured on beef extract-starch or soy agar (Fig. 2A), and not in any other broth or solid media tested (Table S1). To compare this activity with CA production in *S. clavuligerus*, we conducted similar bioassays after growing both organisms on soy agar. Bioactivity in *S. pratensis* plugs was primarily detected when 60 µg/mL of penicillin G was used in the bioassay, compared to the typical 6 µg/mL used for detecting CA production in *S. clavuligerus* (19) (Fig. 2A). Furthermore, a time-course analysis showed that β-lactamase inhibitory activity in *S. pratensis* cultured on soy agar began on day 4 and peaked around day 8 (Fig. 2B). After this, the activity diminished and became undetectable by day 12. In comparison, β-lactamase inhibitory activity in *S. clavuligerus* plugs due to CA production remained detectable beyond day 12. These results suggest that the unknown metabolite produced by *S. pratensis* is either unstable or is synthesized in much smaller quantities at later time points, rendering it undetectable in bioassays. In addition, the inhibition of *E. coli* ESS was not observed in any of the *S. pratensis* cultures tested (Fig. 2A), indicating that the bacterium does not produce any carbapenem antibiotics under the tested conditions.

To determine whether the unknown metabolite produced by *S. pratensis* functions as a β-lactamase inhibitor, metabolomics analysis was performed on methanol extracts from *K. pneumoniae* cultured on bioassay plates containing 60 µg/mL penicillin G, with or without *S. pratensis* plugs (Fig. 2C). In the absence of *S. pratensis* plugs, *K. pneumoniae* growth was not inhibited, and solvent extracts contained benzylpenicilloic acid G, a degradation product indicative of penicillin G hydrolysis by the class A β-lactamase present in *K. pneumoniae* (Fig. S1) (20). Conversely, when *K. pneumoniae* was cultured with *S. pratensis* plugs that resulted in a zone of inhibition, penicillin G remained intact, and benzylpenicilloic acid G was not detected (Fig. 2C). The findings strongly suggest that, like CA (21), the metabolite produced by *S. pratensis* inhibits the β-lactamase from *K. pneumoniae*. This was further demonstrated by performing assays using nitrocefin as a substrate with cell-free preparations of *K. pneumoniae*. Methanol extracts from soy agar cultures of both *S. clavuligerus* and *S. pratensis* inhibited β-lactamase activity, preventing the appearance of the characteristic red color, which is produced on nitrocefin hydrolysis (Fig. 2D).

To further investigate the nature of the β-lactamase inhibitor from *S. pratensis*, high-performance liquid chromatography (HPLC) analysis was conducted on imidazole-derivatized aqueous extracts from soy agar cultures. Similar cultures of *S. clavuligerus* were included in the analysis for comparison. The chromatograms revealed a distinct peak corresponding to CA in *S. clavuligerus*, whereas no such peak was detected in *S. pratensis* extracts (Fig. 2E). Therefore, the results indicate that the β-lactamase inhibitory activity produced by *S. pratensis* is not attributable to CA but is instead due to a different metabolite.

### Transcriptional analysis of the *S. pratensis* CA-like and MM 4550-like gene clusters

**Fig 3:**
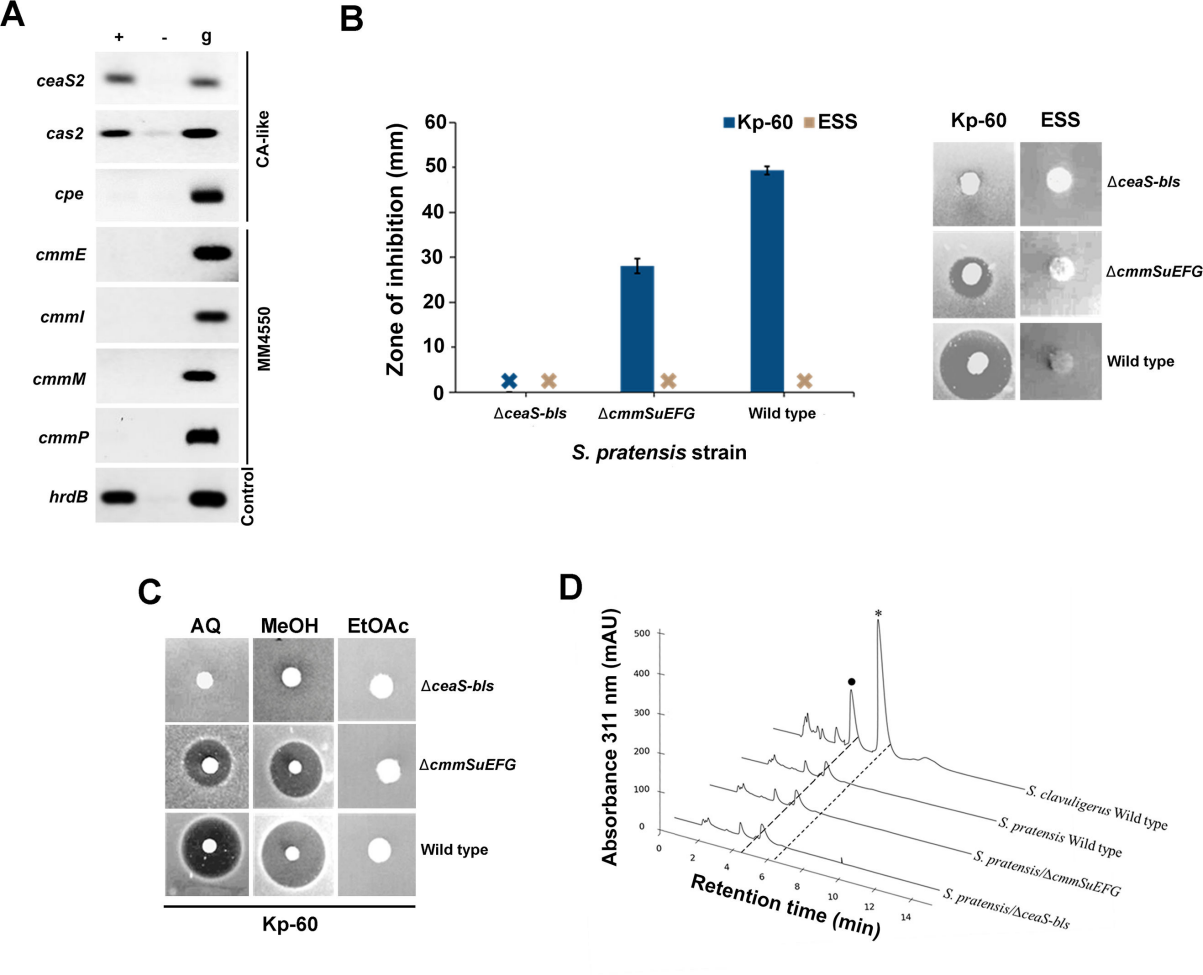
Expression of genes from the CA-like and MM 4550-like biosynthetic gene clusters of *S. pratensis* and phenotypes of corresponding disruption mutants. (**A**) The expression of key genes from the CA-like (*ceaS2*, *cas2*, and *cpe*) and MM 4550-like (*cmmE*, *cmmI*, and *cmmP*) BGCs was analyzed using RT-PCR (+). Isolated RNA that did not undergo reverse transcription was included as a negative control (−). The transcription of the constitutively expressed *hrdB* gene encoding a housekeeping sigma factor was assessed for reference. Identical PCR reactions using isolated genomic DNA as template (**G**) were performed separately to confirm successful amplification. (**B**) Bioactivities of different *S. pratensis* strains cultured on soy agar against *Klebsiella pneumoniae* in the presence of 60 µg/mL penicillin G (Kp-60) and *Escherichia coli* ESS (ESS). The measured zones of inhibition are depicted in the bar graph, with corresponding images of bioassays included as the inset on the right. (**C**) Bioactivities of aqueous (AQ), methanol (MeOH), and ethyl acetate (EtOAc) extracts from different *S. pratensis* strains against *Klebsiella pneumoniae* in the presence of 60 µg/mL penicillin G. (**B and C**) *S. pratensis* strains used: *∆ceaS-bls*, CA-like BGC mutant; *∆cmmEFGH*, MM 4550-like BGC mutant, and the wild-type isolate. (**D**) HPLC analysis of aqueous extracts prepared from different *S. pratensis* strains after imidazole derivatization was conducted using the bondclone method. Extracts prepared from wild-type *S. clavuligerus* cultured under similar conditions were included as a control, where chromatogram peaks corresponding to clavulanic acid (⁕) and 2-hydroxymethyl clavam (•) are indicated.

It was previously reported that when *S. pratensis* was cultured in YEME, R5, TSB, or SA broth for varying durations, only a subset of genes from the CA-like BGC were transcribed and that there was no production of CA or any detectable β-lactamase inhibitory activity (9). The reason for blocked CA production in *S. pratensis* was attributed to the lack of expression of essential genes encoding products involved in the later part of the CA biosynthetic pathway (Fig. 1A) (9). To investigate if the expression of these otherwise silent genes was induced under the agar growth conditions used in the current study, we performed reverse transcription polymerase chain reaction (RT-PCR) analysis on RNA isolated from *S. pratensis* grown for 7 days on soy agar, corresponding to peak production of β-lactamase inhibitory activity (Fig. 2B). Additionally, we also examined the expression of key genes from the *S. pratensis* MM 4550-like BGC to determine if its transcription was somehow activated in our study (Fig. 3A).

The transcription of *cas2* and *ceaS2*, which are part of the CA-like BGC in *S. pratensis* and homologs of which are essential for the early stages of CA production in *S. clavuligerus*, was detected in the *S. pratensis* RNA preparations (Fig. 3A). However, the transcription of *cpe*, a gene required for a later step in the CA biosynthetic pathway in *S. clavuligerus*, was not detected in the same samples (Fig. 3A), suggesting that the final stages of CA biosynthesis are blocked in *S. pratensis*. These results are consistent with those of Alvarez-Alvarez et al. (9), who showed that early genes (*ceaS2*, *oat2*, *oppA1*) are expressed in *S. pratensis* grown in broth culture, while later genes (*cyp*, *cpe*, *orf13*, *orf14*, *oppA2*) remain silent (Fig. 1). Furthermore, there are no paralogues that can compensate for the apparent lack of expression of the genes with undetectable levels of transcription in *S. pratensis*. These results, along with the HPLC analysis described above, provide further support for the hypothesis that the β-lactamase inhibitor produced by *S. pratensis* is not CA.

In the case of the *S. pratensis* MM 4550-like BGC, we examined the expression of *cmmE*, *cmmM*, *cmmP*, and *cmmI* (Fig. 3A). The genes *cmmE* and *cmmM* are homologous to those present in carbapenem producers such as *S. argenteolus* and encode for enzymes that catalyze the first two reactions in the pathway required to form the bicyclic carbapenem core (Fig. 1A and C) (2). Deletion of these genes in other systems abolishes carbapenem production completely (12). Similarly, *cmmP* and *cmmI*, also homologous to genes in *S. argenteolus*, have been shown to be involved in MM 4550 biosynthesis and regulation, respectively. Our analysis revealed that *cmmE*, *cmmM*, *cmmP*, and *cmmI* were not expressed in *S. pratensis* under the tested conditions, indicating that the MM 4550-like gene cluster is silent and is not responsible for producing the detected β-lactamase inhibitory activity. This is supported by the lack of activity of *S. pratensis* plugs against *E. coli* ESS (Fig. 2A), a β-lactam antibiotic-sensitive strain, indicating that the bioactive metabolite is not a carbapenem, including MM 4550 (12).

### Preparation and analysis of *S. pratensis* β-lactam biosynthetic gene cluster mutants

The *S. pratensis* CA-like BGC contains copies of all genes required for CA production in *S. clavuligerus* (Fig. 1B) (9); however, it does not produce the metabolite. Therefore, it is possible that the *S. pratensis* BGC is involved in producing a CA precursor or a novel metabolite with β-lactamase inhibitory activity. Similarly, the *S. pratensis* MM 4550-like BGC also contains copies of all genes responsible for the biosynthesis of MM 4550 in *S. argenteolus* (Fig. 1C) (12), a carbapenem antibiotic from the olivanic acid group that also exhibits β-lactamase inhibition (13). Although our transcriptional analysis suggested that CA-like BGC is partially expressed and that the MM 4550 BGC is silent in *S. pratensis*, low levels of undetectable transcription could potentially result in the production of some bioactive metabolites.

To test this hypothesis, we disrupted genes in *S. pratensis*, homologs of which are essential for producing CA and MM 4550 in *S. clavuligerus* and *S. argenteolus*, respectively. For the CA-like BGC, we simultaneously deleted copies of *ceaS2* and *bls2* (Fig. 1B), which encode gene products responsible for initiating CA biosynthesis and catalyzing the formation of its β-lactam ring in *S. clavuligerus* (4). For the MM 4550-like BGC, we created a *cmmSuEFG* deletion in *S. pratensis* (Fig. 1C), knocking out homologs of genes encoding products involved in the formation and modification of the bicyclic core of MM 4550 in *S. argenteolus* (12). These mutant strains with deletions in each BGC should be completely blocked in the production of any metabolites or intermediates associated with the respective pathways.

The effects of the gene knockouts on the production of the unknown β-lactamase inhibitor were investigated using solid-media fermentation followed by bioassays, HPLC, and liquid chromatography tandem mass spectrometry (LC-MS^2^) analysis. Agar plugs of wild-type *S. pratensis* produced clear zones of inhibition against *K. pneumoniae* in the presence of penicillin G, which were also observed in ∆*cmmSuEFG* mutant (Fig. 3B). It was also noted that the ∆*cmmSuEFG* mutant exhibited reduced growth compared to the wild-type strain on soy agar. In contrast, the activity was completely abolished in the ∆*ceaS-bls* mutant (Fig. 3B), indicating that the β-lactamase inhibitor is a product of the CA-like BGC. Similar results were obtained when bioassays were conducted using aqueous and methanol extracts but not ethyl acetate extracts (Fig. 3C), suggesting that the responsible metabolite is highly polar in nature. Furthermore, no inhibition zones were observed against *E. coli* ESS for any of the plugs or extracts tested (Fig. 3B), indicating that *S. pratensis* does not produce the carbapenem MM 4550 under the tested conditions. In addition, HPLC analysis of imidazole-derivatized aqueous extracts from wild-type *S. pratensis* and the two mutant strains confirmed that none of them produced CA (Fig. 3D).

**Fig 4:**
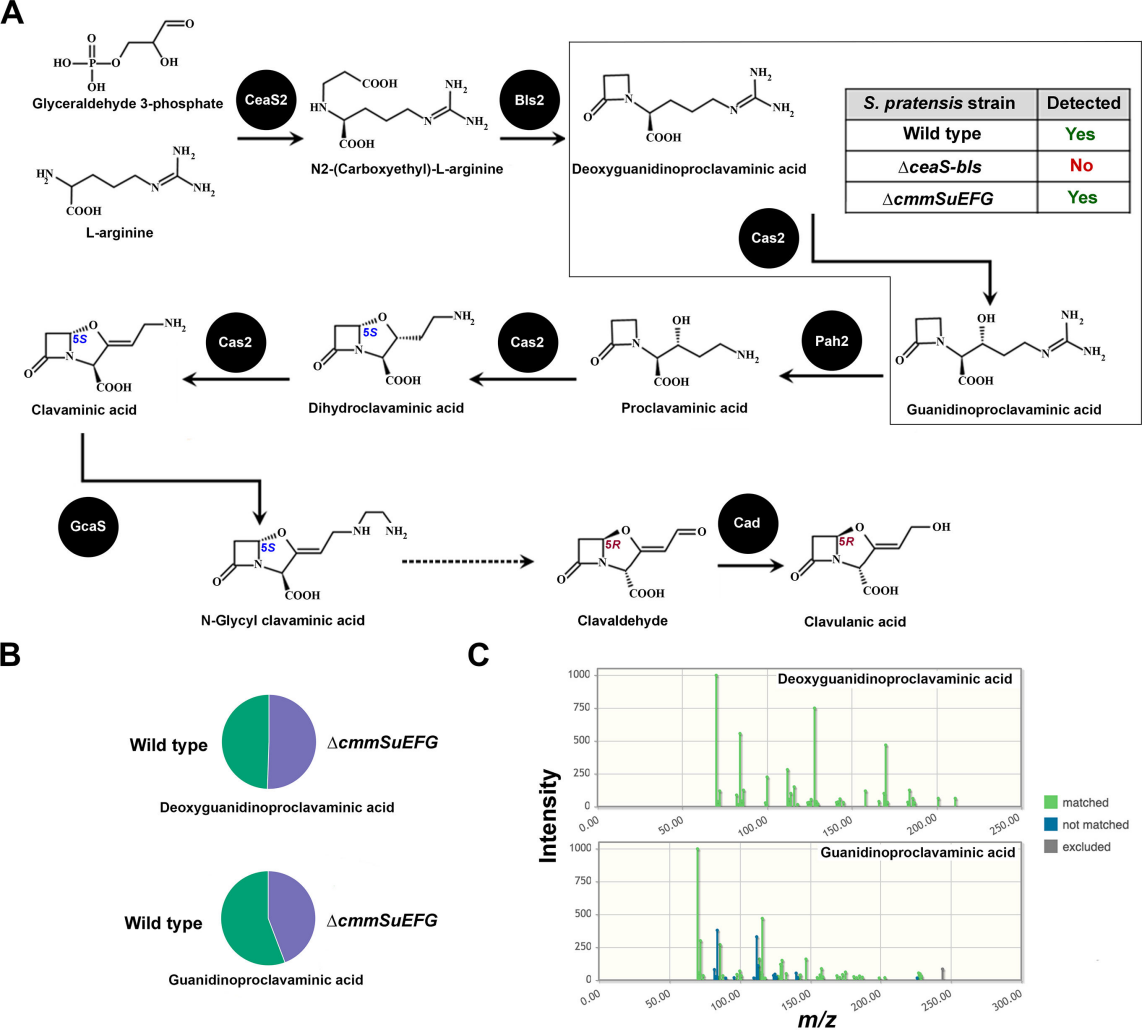
Detection of clavulanic acid precursors in *Streptomyces pratensis* strains using untargeted metabolomics analysis. (**A**) The known pathway leading to clavulanic acid in *Streptomyces clavuligerus* is shown, including the structures of early and some late precursors, along with the identities of the enzymes catalyzing each step (indicated by solid spheres). The gray box highlights two predicted precursors, deoxyguanidinoproclavaminic acid and guanidinoproclavaminic acid, which were detected in wild-type *S. pratensis* and ∆*cmmEFGH* mutants but were absent in the ∆*ceaS-bls* mutant strain. (**B**) The relative amounts of the features corresponding to deoxyguanidinoproclavaminic acid and guanidinoproclavaminic acid detected in wild-type *S. pratensis* (green) and the ∆*cmmEFGH* mutant strain (purple) are shown. Features for the two metabolites were not detected in the *S. pratensis* ∆*ceaS-bls mutant*. (**C**) Correlation between the experimental and predicted MS² fragmentation spectra of the two CA precursors detected in the different *S. pratensis* strains. The legend highlights matched, unmatched, or peaks not considered during the analysis. (**A through C**) *S. pratensis* strains used: ∆*ceaS-bls*, CA-like BGC mutant; ∆*cmmEFGH*, MM 4550-like BGC mutant, and the wild-type isolate.

The loss of β-lactamase inhibitor production in the ∆*ceaS-bls* mutant, coupled with the observation that only early genes from the CA-like BGC are expressed in wild-type *S. pratensis*, suggests that an intermediate from the CA biosynthetic pathway may be responsible for the activity. To further explore this possibility, bioactive methanol extracts from wild-type *S. pratensis* as well as those from the ∆*ceaS-bls* and ∆*cmmSuEFG* strains were subjected to untargeted metabolomics analysis using LC-MS^2^. Compound annotations were performed using GNPS, NAP, and Sirius (22). GNPS and NAP did not predict the presence of any known intermediates or products from the CA or MM 4550 pathways. However, Sirius predicted features corresponding to deoxyguanidinoproclavaminic acid and guanidinoproclavaminic acid, which are early intermediates in the CA biosynthetic pathway (Fig. 4A). The two predicted metabolites were detected in extracts from wild-type *S. pratensis* and the ∆*cmmSuEFG* mutant, but not in the ∆*ceaS-bls* mutant (Fig. 4B). Furthermore, using MetFrag, we successfully matched most of the peaks between the experimental and predicted MS^2^ fragmentation spectra of both metabolites (Fig. 4C). These findings suggest that deoxyguanidinoproclavaminic acid and guanidinoproclavaminic acid, early monocyclic β-lactam precursors of CA, may be responsible for the observed β-lactamase inhibitory activity in *S. pratensis*.

### Examination of β-lactamase inhibitory activity production in *S. clavuligerus* CA-BGC gene mutants blocked at different stages of biosynthesis

In *S. clavuligerus*, deoxyguanidinoproclavaminic acid and guanidinoproclavaminic acid are the second and third intermediates in the CA biosynthetic pathway, formed by the action of the β-lactam synthetase (BLS) and clavaminate synthase (CAS) enzymes (Fig. 4A) (2), respectively. Consequently, disruption of any gene encoding an enzyme catalyzing a reaction downstream of CAS is expected to still permit the production of the two intermediates and retain the associated β-lactamase inhibitory activity if they are responsible for it. To test this hypothesis, we analyzed a set of *S. clavuligerus* strains with gene disruptions that block the CA biosynthetic pathway at different stages.

**Fig 5:**
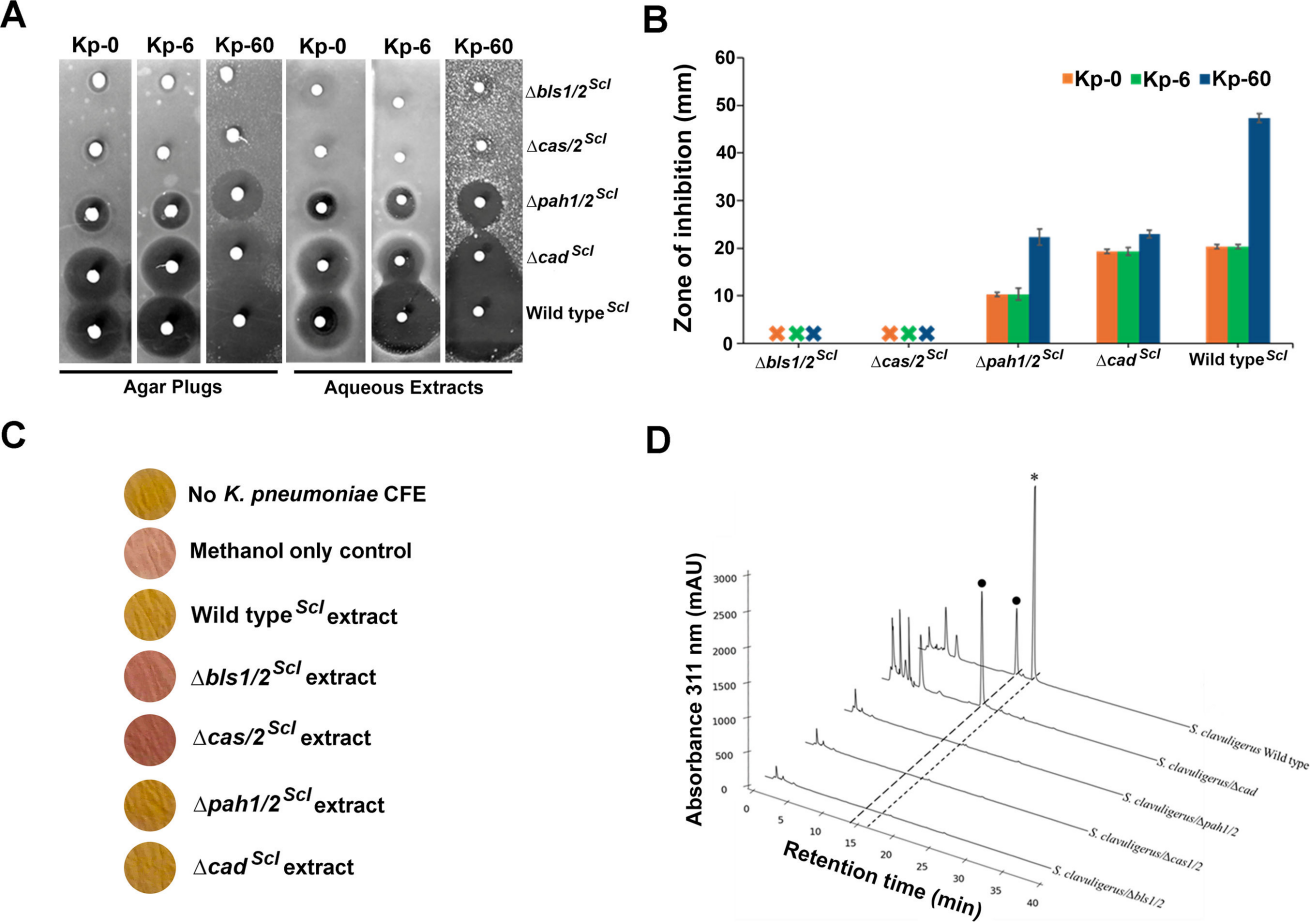
Production of β-lactamase inhibitory activity by *Streptomyces clavuligerus* clavulanic acid biosynthetic gene cluster mutants defective at various stages of biosynthesis. (**A**) Bioactivities of different *S. clavuligerus* strains cultured on soy agar against *Klebsiella pneumoniae* in the absence (Kp-0) or presence of 6 (Kp-6) or 60 (Kp-60) µg/mL penicillin G. Zones of inhibition produced by agar plugs from cultures of different *S. clavuligerus* strains or aqueous extracts prepared using the same cultures are shown. (**B**) The measured zones of inhibition observed in methanol extracts from cultures of different *S. clavuligerus* strains from (**A**) are depicted in the bar graph. (**C**) Detection of β-lactamase inhibition using *K. pneumoniae* CFE with nitrocefin as a substrate. Methanol extracts from soy agar cultures of different *S. clavuligerus* strains, along with appropriate controls (methanol only or those lacking β-lactamase/CFE), are included. Hydrolysis of yellow nitrocefin by β-lactamases produces a characteristic red color, which is inhibited in the presence of a β-lactamase inhibitor. (**D**) HPLC analysis of aqueous extracts prepared from cultures of the respective *S. clavuligerus* strains after imidazole derivatization, assessed using the X-terra method. Peaks corresponding to clavulanic acid (⁕) in the wild-type and 2-hydroxymethyl clavam (•) in the wild-type and Δ*cad^Scl^* mutant strain are indicated. (**A through D**) *S. clavuligerus* strains used: Δ*bls1/2^Scl^*, β-lactam synthetase double mutant; Δ*cas1/2^Scl^*, clavaminate synthase double mutant; Δ*pah1/2^Scl^*, proclavaminic acid amidinohydrolase double mutant; Δ*cad^Scl^*, clavulanic acid dehydrogenase mutant; and the wild-type isolate. The specific steps in the CA biosynthetic pathway catalyzed by the enzymes encoded by the gene for which mutants were assessed are shown in Fig. 4.

Wild-type *S. clavuligerus*, the Δ*bls1/2^Scl^*, Δ*cas1/2^Scl^*, and Δ*pah1/2^Scl^* double mutants, as well as a Δ*cad ^Scl^* mutant (Table S2) (Fig. 4A), were cultured on soy agar and tested for bioactivity against *K. pneumoniae* in the absence and presence of penicillin G (Fig. 5). Because *S. clavuligerus* harbors paralogues of *bls*, *cas*, and *pah*, one in the CA-BGC and the other in either the clavam or paralogue BGCs (which direct the synthesis of 5*S* clavams rather than CA), double mutants of these genes were used to ensure complete blockage of the pathway at the respective enzymatic steps (18). BLS catalyzes the formation of the β-lactam ring of CA in the precursor deoxyguanidinoproclavaminic acid (23), while CAS carries out multiple reactions in the pathway, ultimately leading to the formation of the fused bicyclic β-lactam–oxazolidine ring in clavaminic acid (Fig. 4A) (24). Clavaminic acid is a CA precursor with 5*S* stereochemistry and lacks β-lactamase inhibitory activity (7). Proclavaminate amidinohydrolase (PAH) catalyzes the step immediately following the first CAS reaction in the CA biosynthetic pathway (25), while clavulanic acid dehydrogenase performs the penultimate reaction during CA production in *S. clavuligerus* (Fig. 4A) (4). The results revealed that the Δ*bls1/2^Scl^* and ∆*cas1/2^Scl^* double mutants completely lost the bioactivity in question, and their methanol extracts did not display any *in vitro* β-lactamase inhibitory activity, whereas the ∆*pah1/2^Scl^* and ∆*cad^Scl^* mutants retained both activities (Fig. 5A, B, and C). HPLC analysis of aqueous extracts after imidazole derivatization confirmed that, except for the wild-type strain, none of the *S. clavuligerus* mutants produced CA (Fig. 5D). Since the *S. clavuligerus* ∆*pah1/2^Scl^* and ∆*cad^Scl^* mutants retain activity while the ∆*bls1/2^Scl^* and ∆*cas1/2^Scl^* mutants do not, this suggests that the bioactive intermediate in this case lies within the pathway between the steps catalyzed by CAS and PAH, further pointing to guanidinoproclavaminic acid (Fig. 4A).

### Environmental *Streptomyces* isolates related to *S. pratensis* also produce a β-lactamase inhibitor other than clavulanic acid, sometimes in combination with a potential β-lactam antibiotic

Our work on *S. pratensis* ATCC 33331 (and the *S. clavuligerus* mutants) suggests that an intermediate from the CA biosynthetic pathway might be responsible for the observed β-lactamase inhibitory activity, while the MM 4550 pathway remains silent in this organism. To investigate whether this phenomenon is unique to *S. pratensis* or if the production of a β-lactamase inhibitor distinct from CA also occurs in other organisms, we examined a collection of 10 *Streptomyces* isolates (labeled as JAC 1-10) from our collection. These isolates were selected as their 16S rRNA gene sequences were identical to *S. pratensis*, but they exhibited some variations in their *rpoB* gene sequences (Fig. S2). PCR analysis of genomic DNA revealed that each isolate contained both CA-like and MM 4550-like BGCs (Fig. S3).

**Fig 6:**
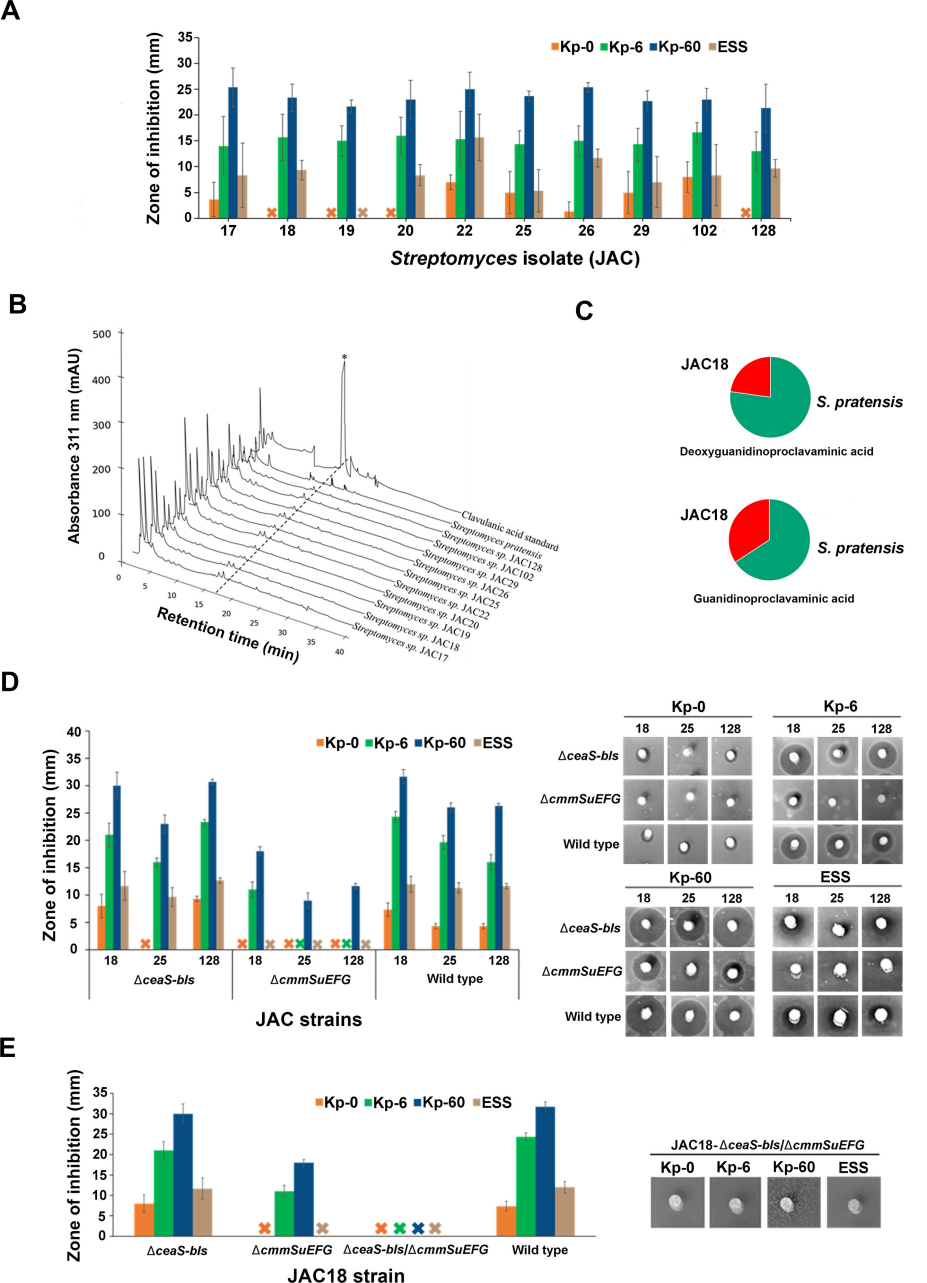
Production of a β-Lactamase inhibitor and potential carbapenem antibiotic by environmental *Streptomyces* (JAC) isolates containing CA-like and MM 4550-like BGCs. (**A**) Bioassays using *Klebsiella pneumoniae* as an indicator in the absence (Kp-0) or presence of 6 µg/mL (Kp-6) and 60 µg/mL (Kp-60) penicillin G to assess β-lactamase inhibitory activity production. *Escherichia coli* ESS was used to detect β-lactam antibiotic activity. (**B**) HPLC analysis of aqueous extracts from different JAC isolates after imidazole derivatization, assessed using the X-terra method. The peak corresponding to clavulanic acid (⁕) in the standard is indicated. (**C**) Relative amounts of the features corresponding to deoxyguanidinoproclavaminic acid and guanidinoproclavaminic acid detected using untargeted metabolomics analysis in wild-type *S. pratensis* (green) and the JAC18 isolate (red). (**D**) Bioactivities of soy agar plugs from wild-type JAC18, JAC15, and JAC128, along with their corresponding mutants defective in the CA-like (∆*ceaS-bls*) and MM 4550-like (∆*cmmEFGH*) BGCs. (**E**) Bioactivities of soy agar plugs from wild-type JAC18 and its corresponding mutants defective in the CA-like (∆*ceaS-bls*), MM 4550-like (∆*cmmEFGH*), or both (∆*ceaS-bls/∆cmmEFGH*) BGCs. (**D and E**) Bioassays were conducted against *K. pneumoniae* in the absence (Kp-0) or presence of penicillin G (Kp-6, 6 µg/mL; Kp-60, 60 µg/mL), as well as against *E. coli* ESS (ESS). The measured zones of inhibition are depicted in the bar graph, with corresponding bioassay images included as insets on the right.

The 10 JAC isolates were cultured on soy agar, and bioassays using plugs from all of them displayed activity against *K. pneumoniae* in the presence of penicillin G (6 and 60 µg/mL) (Fig. 6A), indicating β-lactamase inhibitor production. *Streptomyces* isolates JAC17, JAC22, JAC26, and JAC102 also inhibited the growth of *K. pneumoniae* in the absence of penicillin G (Fig. 6A). Additionally, all of them, except for JAC19, exhibited activity against *E. coli* ESS (Fig. 6A), indicating possible β-lactam antibiotic production in these strains. However, aqueous and methanol extracts from the same agar cultures exhibited only β-lactamase inhibitory activity, suggesting that the carbapenem may not be extractable or that another metabolite could be responsible for the observed activity against *E. coli ESS*. Notably, in *S. argenteolus*, carbapenem production by the wild-type strain occurs at very low levels, also making extraction and detection of MM 4550 particularly challenging (12). HPLC analysis of aqueous extracts also showed that, like *S. pratensis*, none of the JAC isolates produced detectable levels of CA (Fig. 6B). Since methanol extracts from the strains also displayed β-lactamase inhibition, one of them (from JAC18) was subjected to untargeted LC-MS^2^ analysis as described earlier. Metabolomics analysis revealed the presence of features corresponding to deoxyguanidinoproclavaminic acid and guanidinoproclavaminic acid (Fig. 6C), but not CA or any of the other intermediates in the JAC18 extract. In addition, it appears that the relative level of production of the two metabolites is much lower in JAC18 as compared to *S. pratensis* (Fig. 6C). Overall, these results suggest that at least one independently isolated *Streptomyces* strain related to *S. pratensis*, specifically JAC18, and potentially others, produce CA precursors and β-lactamase inhibitory activity, indicating that this phenomenon may be more widespread. Additionally, some isolates also produce a carbapenem or another metabolite capable of inhibiting *E. coli ESS* (12).

### Genome sequencing of select JAC isolates and analysis of β-lactam biosynthetic gene cluster mutants

Three JAC isolates (JAC18, JAC25, and JAC128) were selected based on differences in bioactivity production profiles (Fig. 6A) for paired-end Illumina sequencing, and the genome of *S. pratensis* was re-sequenced for comparison (Table S3). Analysis of the assembled genomes using AntiSMASH (26) revealed that all BGCs identified in *S. pratensis* were also present in the three sequenced JAC isolates (Table S4). Additionally, some JAC isolates contained one or more gene clusters not found in *S. pratensis* (Table S5). Since JAC18, JAC25, and JAC128 possess β-lactam BGCs identical to those in *S. pratensis*, we investigated whether the β-lactamase inhibitory and antibiotic activities observed in some of these isolates could be attributed to the CA-like or MM 4550 BGCs, respectively. Knockout strains of the three isolates defective in each of these BGCs were generated using the same plasmids that we previously used to prepare the corresponding *S. pratensis* disruption mutants (Table S6).

Bioactivity production in different strains was assessed after solid media fermentation as previously described (Fig. 6D). Plugs from parental JAC18, JAC25, and JAC128 wild-type strains inhibited the growth of *K. pneumoniae* (with and without penicillin G) and *E. coli* ESS. In contrast, the ∆*cmmSuEFG* (carbapenem MM 4550-like) knockout strains exhibited inhibition only against *K. pneumoniae* in the presence of penicillin G, with no activity observed against *E. coli* ESS (Fig. 6D). These findings indicate that the production of any potential carbapenem antibiotic was blocked in the JAC18, JAC25, and JAC128 ∆*cmmSuEFG* mutants, whereas the β-lactamase inhibitor associated with the intact CA-like BGC was still produced in them.

When compared to the respective parental wild-type strains, the ∆*ceaS-bls* (CA-like BGC) mutants of JAC18, JAC25, and JAC128 did not display any significant differences in bioactivities against *K. pneumoniae* (with or without penicillin G) or *E. coli* ESS (Fig. 6D). This residual bioactivity in the ∆*ceaS-bls* mutants can be attributed to the presence of an intact MM 4550-like BGC in them, as MM 4550 or olivanic acids are known to function as both antibiotics and β-lactamase inhibitors (12). In addition, HPLC analysis of aqueous extracts confirmed that none of the wild-type or mutant strains tested produced CA. These results indicate that JAC18, JAC25, and JAC128 potentially produce both a carbapenem and a β-lactamase inhibitor, which is not CA.

To link the observed bioactivity production phenotypes with specific BGCs, we generated a ∆*ceaS-bls/*∆*cmmSuEFG* double knockout strain (defective in both the CA-like and MM 4550-like BGCs) of JAC18. The double mutant strain did not display any inhibitory activity against *K. pneumoniae* (with or without penicillin G) or *E. coli* ESS under the tested conditions (Fig. 6E). Taken together, the results demonstrate that the β-lactam and β-lactamase inhibitory activities observed in JAC18 on soy agar are attributable to the MM 4550-like and CA-like BGCs. By extension, a similar mechanism may explain the activities seen in other JAC isolates from this study, although further analysis is needed to draw definitive conclusions.

### The link between CA-like and carbapenem BGCs and its possible implications

**Fig 7:**
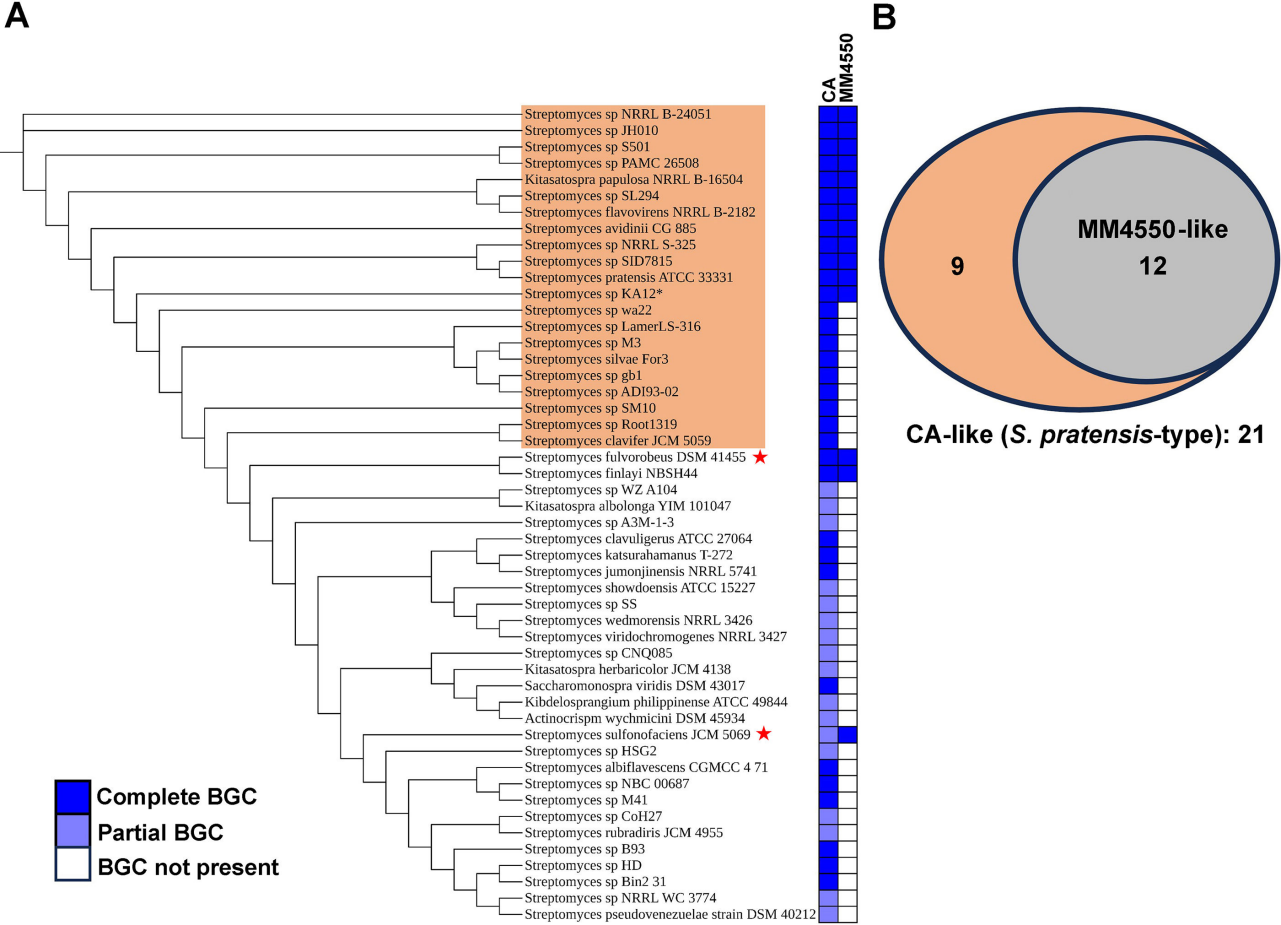
Co-occurrence of CA-like and carbapenem BGCs in bacterial genomes present in the NCBI database. (**A**) The core genome phylogenetic tree depicts the relationships between the organisms shown, with different colored boxes indicating the presence of complete or partial BGCs, or their absence. Isolates containing the CA-like BGC from *S. pratensis* are highlighted with an orange box, whereas those that also contain the cephamycin C BGC along with the two other types of gene clusters are marked with red stars. (**B**) The Venn diagram illustrates the number of isolates containing the MM 4550-like (gray inner circle) and CA-like BGCs from *S. pratensis*, which correspond to the orange box depicted in A.

Overall, our results indicate that most isolates possessing both MM 4550-like and CA-like BGCs produce β-lactam antibiotic and β-lactamase inhibitory activities (Fig. 6). Such phenomena have been noted before in *Streptomyces* species, such as *S. clavuligerus*, *S. jumonjinensis*, and *S. katsurahamanus*, which produce both cephamycin C and CA (4, 6). In *S. pratensis*, JAC18, and possibly other JAC isolates, the β-lactamase inhibitor is not CA itself, but rather a predicted bioactive precursor. Additionally, the β-lactam antibiotic produced by JAC18, JAC25, JAC128, and possibly other isolates is most likely the carbapenem MM 4550 or a related metabolite, which also exhibits β-lactamase inhibitory activity (12, 13). This is evident from the bioactivities detected against *K. pneumoniae* and *E. coli* ESS in the different wild-type JAC isolates and the corresponding CA-like BGC mutants prepared in the current study. In contrast, the MM 4550-like BGC disruption mutants of *S. pratensis* and the JAC isolates display only β-lactamase inhibition, linking this residual activity to the CA-like BGC and the possible precursors produced in them (Fig. 3 and 6). Most importantly, it was demonstrated that the JAC18 double knockout strain, with deletions in both the CA-like and MM 4550-like BGCs, exhibits no β-lactamase inhibitory or β-lactam antibiotic activity at all (Fig. 6). The presence of thienamycin (a carbapenem) and cephamycin C BGCs, along with the production of these two unrelated β-lactam antibiotics, has been previously reported in *Streptomyces cattleya* (11). We previously noted the presence of certain β-lactam biosynthetic genes from different families in diverse organisms (6, 27), and in the current study, we isolated *Streptomyces* containing the CA-like and MM 4550-like BGCs from the environment. These findings raise further questions regarding the co-occurrence of the two BGCs in bacteria in general. Therefore, we used Cblaster (28) to query the NCBI database with the sequences of CA-like and MM 4550-like BGCs from *S. pratensis* separately, which led to the identification of 50 organisms containing different variants of either one or both BGCs (Fig. 7). It is important to note that some of the genomes examined were fragmented, and in such instances, the BGCs identified might not be complete and could be missing peripheral genes.

Many distinct variants of the CA-like BGC were identified during the analysis (Fig. S4), some of which match the ones present in *S. pratensis* and *S. clavuligerus*, respectively. Twenty-one organisms contained *S. pratensis*-type CA-like BGCs with the same gene content and arrangement, and 12 of them also harbored carbapenem BGCs (Fig. 7), which were identical to the MM 4550-like BGC from *S. pratensis*. We also observed the co-occurrence of three other variants of the two types of BGCs, as seen in the case of *Streptomyces sulfonofaciens* JCM 5069 (Fig. S5), which is known to produce a different olivanic acid metabolite than MM 4550 (29). *S. argenteolus* was not included in the analysis due to its incomplete genome sequence, preventing the examination of β-lactam BGC co-occurrence. Notably, both the MM 4550-like and CA-like BGCs from *S. pratensis* are frequently found in different *Streptomyces* (Fig. 7B). We also analyzed the genomes of the 50 organisms for the presence of other β-lactam BGCs and found that two of them, *Streptomyces fulvorobeus* DSM 41455 and *S. sulfonofaciens* JCM 5069, contain CA-like, carbapenem, and cephamycin C BGCs (Fig. 7). This co-occurrence of three β-lactam BGCs within a single organism is a phenomenon not previously reported.

The carbapenem BGCs found in organisms containing CA-like BGCs are, in most instances, predicted to be of the olivanic acid (mostly MM 4550) and not the thienamycin type (Fig. 7; Fig. S5). The biosynthesis of MM 4550 and thienamycin is proposed to follow a similar strategy during the early stages of production before diverging, resulting in the stereochemical inversion of the C-6 and C-8 stereocenters and the addition of a sulfate group in MM 4550 (Fig. 1A) (12). The absence of CA production in JAC isolates harboring both clavam and carbapenem BGCs suggests that the CA-like BGCs present in these strains are not fully active under the conditions tested (Fig. 6A and B). The production of a potent β-lactamase inhibitor like CA in organisms with MM 4550-like BGCs is unlikely to provide a significant advantage, as these organisms already have the capacity to produce olivanic acid carbapenems, which possess both antibiotic and β-lactamase inhibitory activity. Alternatively, the β-lactamase inhibitors produced by these organisms may have distinct activity spectra or synergistic effects, which could provide some selective advantage. Many synthetic monobactams with diverse structures have been shown to exhibit a wide range of antibacterial and some β-lactamase inhibitory properties (30). The co-production of monobactam-like intermediates from the CA biosynthetic pathway alongside olivanic acids in certain organisms may indicate potential synergism, analogous to what is observed in CA and cephamycin C co-producers in nature (5, 6). If this is indeed the case, then combinations of different monobactams with olivanic acids or other carbapenems warrant further attention to investigate their potential for therapeutic applications (31, 32).

Evidence suggesting that early intermediates in the CA biosynthetic pathway, prior to the formation of the bicyclic 5*S* clavam core in *S. pratensis* and *S. clavuligerus* (Fig. 4 and 5), retain β-lactamase inhibitory activity is novel. This activity is apparently lost in the 5*S* clavam precursors during CA biosynthesis in *S. clavuligerus* but is subsequently regained following the establishment of 5*R* stereochemistry in clavaldehyde and clavulanic acid, possibly with more potency (Fig. 4 and 5) (33). Many CA-like BGCs with diverse arrangements have been identified in bacterial genome sequences (6). It would be interesting to investigate whether these BGCs produce either bioactive intermediates, CA, or perhaps another yet unidentified clavam metabolite, the latter of which could also be the case for the fully active gene cluster from *S. pratensis*. In some of our previous work, we demonstrated that orthologues of late-stage genes from CA-like BGCs from non-producers, including *S. pratensis*, could not complement mutants of *S. clavuligerus* defective in corresponding genes, demonstrating that the encoded proteins are not functionally equivalent (34). These findings suggest that the lack of expression of certain genes from CA-like BGCs in organisms such as *S. pratensis* may not fully account for the absence of CA production. It has also been noted that CA production only occurs in organisms that contain the cephamycin C BGC as part of a β-lactam supercluster in conjunction with a CA BGC (6). Therefore, one possibility is that the *S. pratensis* and other singleton CA-like BGCs represent ancestral forms of the CA BGCs found in producers such as *S. clavuligerus*, reflecting different stages of their evolutionary trajectories. A search for DNA sequences in the public database using the *S. clavuligerus* CA BGC revealed that less than one-third of the hits contained a full set of homologous genes, while more than half included genes necessary for synthesizing the early CA precursors predicted in the current study (Fig. S6). It can be hypothesized that CA production evolved after the acquisition of fully assembled BGCs alongside those responsible for producing other conventional β-lactam antibiotics, such as cephamycin C. This seems to be the case for *S. clavuligerus*, *S. jumonjinensis*, and *S. katsurahamanus* (5, 6), where the evolutionary advantage to the producer is evident (35). Testing this hypothesis will require a detailed phylogenetic analysis to reconstruct the evolutionary relationships and pathways associated with the different CA-like BGCs.

To conclude, the co-occurrence of CA-like and carbapenem BGCs, and possibly those from other β-lactam subfamilies, suggests that the co-production of these metabolites may occur more frequently in natural environments than previously recognized. Moreover, a BGC that appears to be silent in one isolate, as observed with the MM 4550-like BGC in *S. pratensis*, may be active in other closely related strains. This was evident from the analysis conducted on the JAC isolates, which consistently exhibited bioactivities associated with the CA-like and MM 4550-like BGCs in the current study. These findings emphasize the evolutionary flexibility and functional diversity of these biosynthetic pathways across related bacterial strains. This study also highlights the need for further investigations into the ecological roles, distribution, and co-occurrence of β-lactam BGCs. Addressing these questions remains a long-term research priority for our group.

## MATERIALS AND METHODS

### Bacterial strains, plasmids, media, and culture conditions

All media and reagents were obtained from Fisher Scientific, VWR International, or Sigma-Aldrich (Canada). Information on the bacterial strains and plasmids used in this study, along with their sources, is summarized in Tables S2 and S6, respectively. *Streptomyces* species and isolates were cultured on agar plates and in broth following established methods (18, 36). For bioactivity analysis, *S. pratensis* ATCC 33331 was cultured under different nutritional conditions (Table S1). For routine analysis, 100 µL of *Streptomyces* spore stocks were plated on agar media and incubated for up to nine days to prepare plugs and extracts for testing (36). All *Streptomyces* cultures were maintained at 28°C, liquid cultures were agitated at 250 rpm, and further manipulations were conducted using standard procedures (37). When necessary, antibiotic supplements were added to cultures of mutant strains or those harboring plasmids (38). *Escherichia coli* cultures were grown and maintained according to standard protocols (39).

### Soy agar sample preparation, bioassays, and HPLC analysis

*Streptomyces* strains were streaked onto soy agar and incubated at 28°C for 7 days in the case of *S. pratensis* and the JAC isolates or 9 days for *S. clavuligerus* (36). Agar plugs were prepared for direct testing using a sterilized 9 mm cork borer. For aqueous extract preparation, up to six agar plugs were placed in a 1.5 mL Eppendorf tube and frozen at −80°C for at least 1 hour. The tubes were then centrifuged at high speed for 10 minutes, and the aqueous supernatant was collected for further analysis. Methanol extracts were prepared as described previously (6).

Bioassays were conducted using previously described procedures (6). β-lactamase inhibition was assessed using *Klebsiella pneumoniae* ATCC 15380 with 6 or 60 µg/mL penicillin G, and controls without the antibiotic were included for comparison. Additionally, *Escherichia coli* ESS was employed to detect the production of β-lactam antibiotics, including cephamycin C and carbapenems (12). Agar plugs were either placed directly onto bioassay plates or aqueous/methanol extracts were spotted onto sterilized paper discs, as described previously (18).

HPLC analysis of imidazole-derivatized supernatants and extracts was performed using two different methods. The analyses employed either a Bondclone C18 column (100 × 8 mm, 10 µm, 148 Å, Phenomenex, USA) or an Xterra column (2.1 × 150 mm, 3.5 µm, 125 Å, Waters Scientific, USA) to detect the production of clavulanic acid and related metabolites, as described previously (6, 34).

### Analysis of DNA, RNA, and general molecular methods

Standard protocols were used for DNA isolation and manipulation from *E. coli* (39) and *Streptomyces* species (37). Total RNA was extracted from *S. pratensis* grown on cellophane discs placed on soy agar as described previously (34). Reverse transcription (RT) was performed using the Maxima H Minus First Strand cDNA Synthesis Kit (Thermo Scientific, USA). PCRs were carried out using the Phusion or Taq DNA polymerase kits (ThermoFisher, USA). When necessary, PCR products were cloned into the pGEM-T Easy vector (Promega, USA) following the manufacturer’s instructions, and Sanger sequencing was performed at the Center for Applied Genomics, University of Toronto (Canada) for verification of all inserts. All DNA oligonucleotide primers (Table S7) were purchased from Integrated DNA Technologies (USA).

The 16S rRNA gene was amplified using universal primers (Table S7), while *rpoB* gene sequences for the 10 JAC isolates were obtained from Liu et al. (40). Sequence alignments for both the 16S rRNA and *rpoB* genes were performed using ClustalW in MEGA (41). Genomic DNA PCR was carried out to confirm the presence of essential genes from the CA-like (*ccaR*, *car*, *ceaS2*, and *gcas*) and MM 4550-like (*cmmE, cmmI*, *cmmP*, and *cmm17*) BGCs in the JAC isolates. The primers used (Table S7) were designed based on the *S. pratensis* genome sequence.

### Preparation of the ∆*ceaS-bls,* ∆*cmmEFGH, and* ∆*ceaS-bls/*∆*cmmEFGH* mutant strains

The pTOPO PCR Cloning Kit (Invitrogen, Canada) was used to prepare knockout vectors targeting the CA-like and MM 4550-like BGCs in *S. pratensis* and the three JAC isolates (Table S2). First, the apramycin resistance (*apra*) cassette along with *oriT* from pIJ773 (42) was PCR-amplified using primers containing *NdeI* restriction sites at their 5′ ends (Table S7). The amplified fragment was ligated into the pGEM-T Easy vector following the manufacturer’s instructions (Promega, USA), resulting in pGEMT-*apra-oriT*. This plasmid served as a source of the *apra-oriT* cassette as an *NdeI* fragment for subsequent gene deletion and disruption studies (Table S6).

To target the CA-like BGC, 3 kb DNA fragments from the regions immediately upstream (U) and downstream (D) of *ceaS* and *bls* in *S. pratensis* were amplified by PCR using primers with engineered restriction sites (Table S7). The upstream fragment was cloned into pTOPO to generate pTOPO-clav/U, incorporating *NotI*/*NdeI* flanking sites, while the downstream fragment was cloned into pTOPO to create pTOPO-clav/D with engineered *NdeI*/*EcoRV* flanking sites (Table S6). Clones with inserts in the correct orientation were identified by restriction digestion and verified by sequencing. The plasmid pTOPO-clav/D was digested with *NdeI* and *XbaI* to isolate the downstream insert, which was subsequently ligated into the similarly digested pTOPO-clav/U, resulting in pTOPO-clav/UD. This construct was then linearized with *NdeI*, and the *apra-oriT* fragment from pTOPO-*apra-oriT* was inserted to generate the gene disruption plasmid, pTOPO-clav/UAD.

The MM 4550-like BGC targeting construct, pTOPO-carb/UAD, was prepared using the same strategy as described for pTOPO-clav/UAD, except that four genes, *cmmEFGH*, were selected for deletion. The upstream and downstream PCR products were flanked by *HindIII*/*NdeI* and *NdeI*/*SpeI* restriction sites, respectively. Additionally, the downstream fragment was initially cloned into pGEM-T Easy before being transferred to pTOPO-carb/U to give pTOPO-carb/UD (Table S6).

The disruption constructs pTOPO-clav/UAD and pTOPO-carb/UAD were individually introduced into *S. pratensis*, JAC18, JAC25, and JAC128 by conjugation using *E. coli* ET12567/pUZ8002, following previously described methods (18). Apramycin-resistant ex-conjugants were selected and induced to sporulate to promote double crossover events. Mutant strains that were apramycin-resistant (gene disruption marker) and kanamycin-sensitive (loss of the pTOPO vector) were isolated and confirmed by PCR analysis of purified genomic DNA. This led to the *S. pratensis*, JAC18, JAC25, and JAC128 *∆ceaS-bls* and *∆cmmEFGH* single mutants used in the current study (Table S2).

To prepare the JAC18 ∆*ceaS-bls*/∆*cmmEFGH* double mutant, a plasmid containing the hygromycin resistance cassette, pGEMT-*hyg-oriT*, was first constructed by PCR amplifying the insert from plasmid pIJ10700 (42) using primers with *NdeI* restriction sites at their 5′ ends (Table S7). The amplified fragment was ligated into the pGEM-T Easy vector following the manufacturer’s instructions (Promega, Canada). Following this, the *apra-oriT* cassette in pTOPO-clav/UAD (Table S6) was replaced with the hygromycin cassette from pGEMT-*hyg-oriT* using the flanking *NdeI* sites introduced earlier. This resulted in pTOPO-clav/UHD, which was subsequently introduced into the JAC18 ∆*cmmEFG* single mutant strain via conjugation. Apramycin- and hygromycin-resistant ex-conjugants were selected, and the ∆*ceaS-bls*/∆c*mmEFG* double mutant strain was isolated, which was subjected to whole-genome sequencing for confirmation.

### Liquid chromatography tandem mass spectrometry analysis

To test for *in vivo* β-lactamase inhibition, plugs from soy agar plates of *S. pratensis* were used in bioassays employing *K. pneumoniae* ATCC 15380 (Table S2) in the presence of penicillin G (60 µg/mL). After overnight incubation, the plugs, along with cores from the region below where the growth of *K. pneumoniae* was inhibited, were collected. Corresponding cores from *K. pneumoniae* plates that did not receive *S. pratensis* plugs, and where no growth inhibition occurred, were also collected. One hundred such cores were extracted with 10 mL of methanol, and the extracts were concentrated and analyzed by LC-MS^2^ as previously described (22). Methanol extracts prepared from agar cultures of wild-type *S. pratensis*, the ∆*ceaS-bls* and ∆*cmmEFG* mutants, as well as the JAC18 isolate, were analyzed using the same LC-MS^2^ methodology, except that an Accucore Polar Premium C18 column (2.1 mm × 150 mm × 2.6 µm, Thermo Fisher, USA) was used for LC separation.

Analysis of untargeted LC-MS^2^ data from extracts of *K. pneumoniae* cultures to monitor β-lactamase activity was conducted using GNPS (43) as described previously (22). Feature-based molecular networking in GNPS (44) was used for analyzing data from the different *S. pratensis* strains and the JAC18 isolate in the current study, using previously defined parameters (45). For comparison of MS^2^ spectra in MetFrag (46), background peaks with an area below 1.5e5 were filtered.

### Genome sequencing, gene cluster identification, and bioinformatics analyses

Genomic DNA was isolated from *Streptomyces* strains using the Presto Mini gDNA Bacteria Kit (Geneaid Biotech Ltd., Taiwan). Illumina paired-end sequencing was performed as previously described (47). For genome assembly, raw sequencing data in FastQ format were initially preprocessed using Fastp (48) and then assessed for quality as described previously (49). Taxonomic classification of the DNA sequences was performed using Kraken2 v2.1.2 (50). Both *de novo* and reference-based assembly of the preprocessed reads were conducted using SPAdes (51), which were further improved through scaffolding with RagTag (52), and quality was evaluated using QUAST (53). This Whole Genome Shotgun project PRJNA1222421 has been deposited at DDBJ/ENA/GenBank (https://www.ncbi.nlm.nih.gov, *S. pratensis* ATCC 33331: JBPGVX000000000; JAC18: JBNHTM000000000; JAC25: JBNHTL000000000; and JAC128: JBNHTK000000000).

Whole-genome sequences of *S. pratensis* ATCC 33331, JAC18, JAC25, and JAC128 were analyzed using the antiSMASH 6.0 web server using default parameters to annotate specialized metabolite BGCs (26).

Co-occurrence analysis of CA-like and the carbapenem MM 4550-like BGCs was performed using sequences of the respective BGCs from *S. pratensis* to conduct BLAST searches against the NCBI database using cblaster along with default settings in February 2024 (28). Only BGCs with associated genome sequences were retained for analysis to detect the presence of other β-lactam clusters. Alignments were generated and visualized using cblaster, and BGCs missing core genes required for producing the two types of metabolites were filtered out as they most probably represent distant lineages. The genomes of organisms containing BGCs homologous to those identified in *S. pratensis* were downloaded from the NCBI database and annotated using Prokka (54) with default settings. The resulting GFF files were used in Roary (55) on the command line to construct a core-genome phylogenetic tree. The tree was visualized using iTOL (56), with annotations indicating the presence of the respective BGCs in each genome/organism. The presence of additional β-lactam BGCs in the organisms containing CA-like and the carbapenem MM 4550 BGC was analyzed using antiSMASH 6.0 (26). For the analysis of CA-like BGCs with varying coverage or gene content, the same clustering strategy was used to query the entire database in November 2024, including sequences of BGCs and genomes. The CA-BGC from *Streptomyces clavuligerus* ATCC 27064 was used as the query (maximum e-value: 0.01; minimum identity: 30%; minimum query coverage: 50%; minimum unique query hits: 2; minimum hits in cluster: 2). Duplicate hits were then removed before compiling the data.

## SUPPLEMENTAL MATERIAL

Supplemental Figures and Tables msphere.00188-25-s0001.pdf Figures S1-S6 and Tables S1-S7.

ASM does not own the copyrights to Supplemental Material that may be linked to, or accessed through, an article. The authors have granted ASM a non-exclusive, world-wide license to publish the Supplemental Material files. Please contact the corresponding author directly for reuse.
