# Supplementary material for: Functional insights into Streptomyces isolates containing both clavulanic acid-like and carbapenem biosynthetic gene clusters
Source: mSphere. 2025 Aug 25;10(9):e00188-25. doi: 10.1128/msphere.00188-25 (PMC12482188; doi:10.1128/msphere.00188-25)
Supplement: Supplemental Figures and Tables — Figures S1-S6 and Tables S1-S7. [file msphere.00188-25-s0001.pdf]

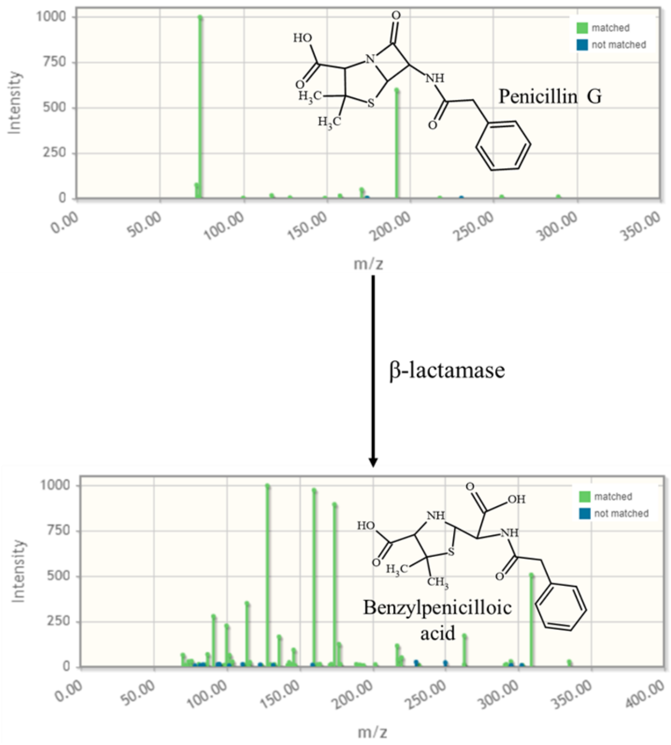

Supplementary Figure S1: Comparison plots from MetFrag showing mass spectrometry (MS^2) fragmentation patterns of penicillin G and its β-lactamase-degraded product, benzylpenicilloic acid, detected in *Klebsiella pneumoniae* ATCC 15380 cultures containing penicillin G in the presence or absence of *Streptomyces pratensis* ATCC 33331 plugs, respectively. Shared peaks between predicted and experimental spectra are highlighted in green, while unmatched peaks are shown in blue. These comparisons provide insight into the degradation of penicillin G by β-lactamase and the potential inhibition of this process by metabolites produced by *S. pratensis*.

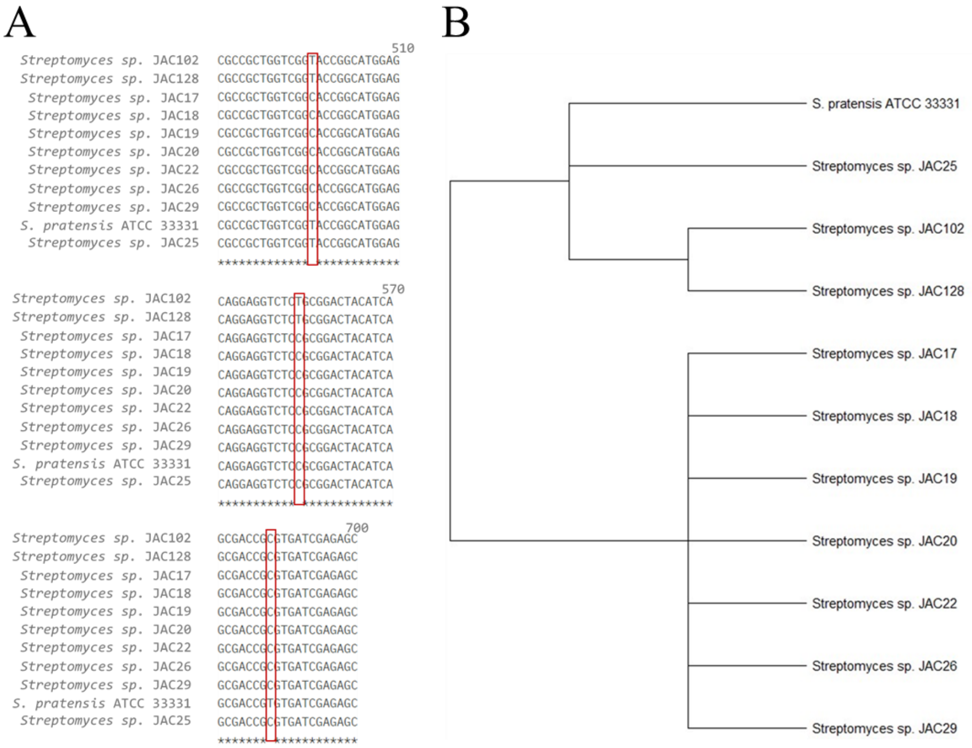

Supplementary Figure S2: Comparison of *rpoB* gene DNA sequences between *S. pratensis* ATCC33331 and the ten JAC *Streptomyces* environmental isolates from the current study. **(A)** Multiple nucleotide sequence alignment of selected regions of *rpoB* where differences were observed between *S. pratensis* and ten JAC isolates (red box). The numbers at the top indicate nucleotide positions in the alignment. (**B**) Phylogenetic tree illustrating the genetic relationships among the analyzed *Streptomyces* species based on *rpoB* sequences. The tree was constructed using the Maximum Likelihood method in MEGA 11 (https://www.megasoftware.net/).

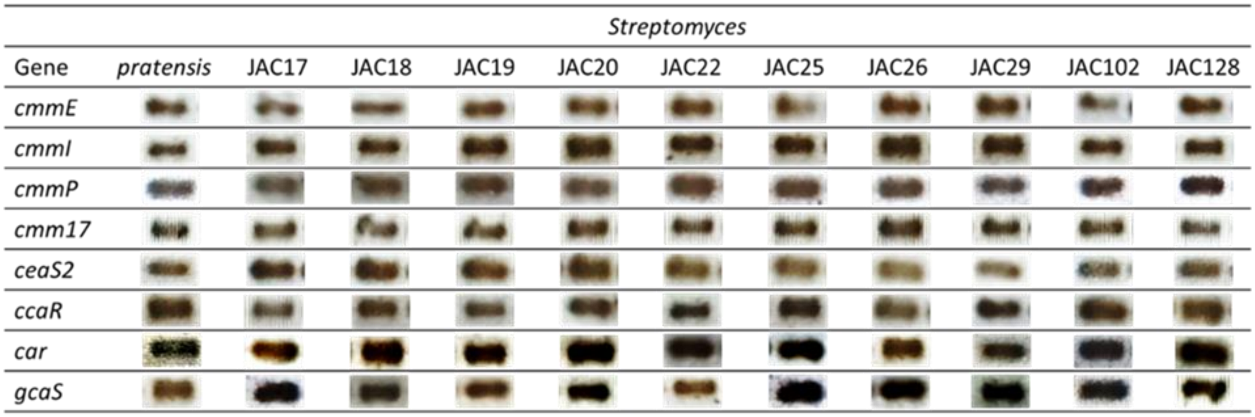

Supplementary Figure S3: Use of genomic DNA PCR to detect the presence of early, middle, and late genes from clavulanic acid-like and carbapenem MM4550-like BGCs in the ten *Streptomyces* JAC isolates from the current study. Genomic DNA PCR was performed to assess the presence of genes associated with the respective BGCs and *S. pratensis* was included as a positive control. The carbapenem BGC genes from *S. pratensis* investigated were *cmmE*, *cmmI*, *cmmP*, and *cmm17*, while the clavulanic acid BGC genes included *ceaS2*, *ccaR*, *car* (or *cad*), and *gcaS*. PCR amplification using primers specific for each gene confirmed the presence of both BGCs in all JAC isolates tested.

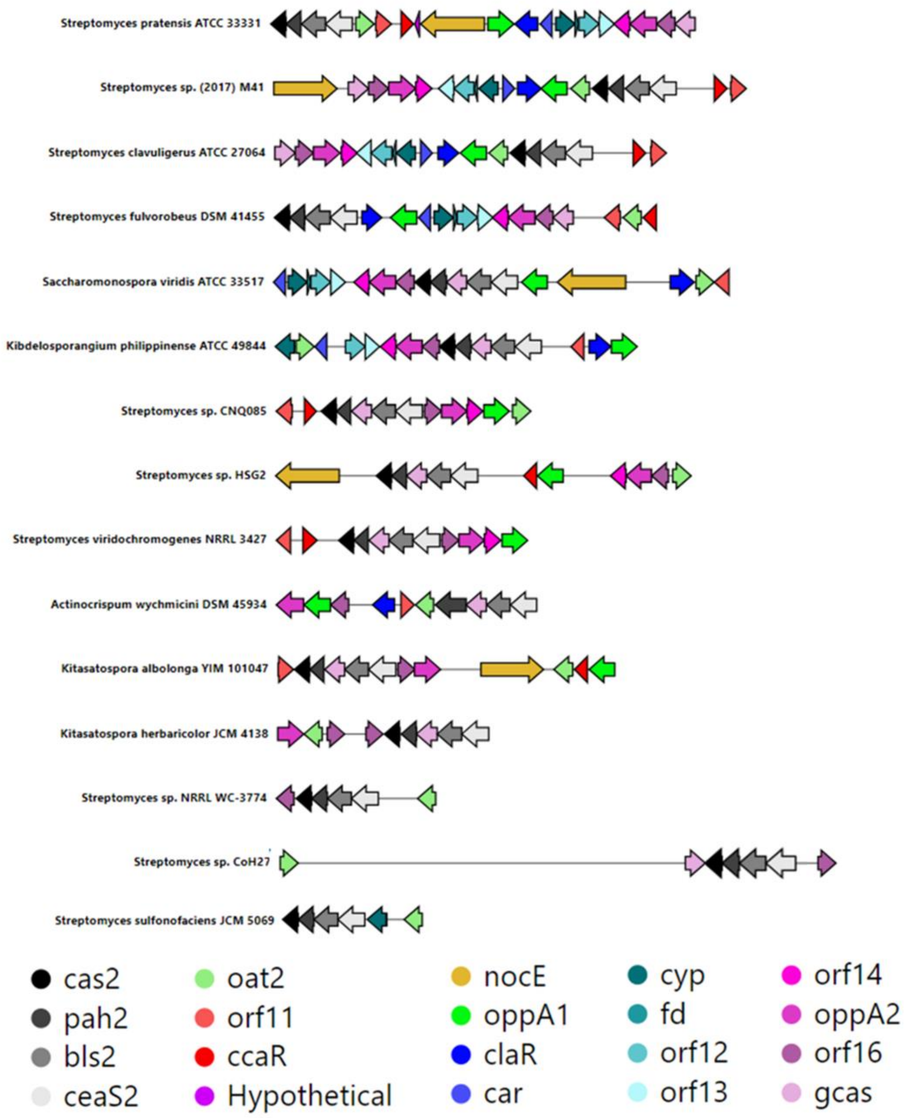

Supplementary Figure S4: Gene alignment of representative clavulanic acid-like BGCs from publicly available genomes from the NCBI database. The analysis was conducted using cblaster, a bioinformatics tool designed for comparative BGC analysis in February 2024.

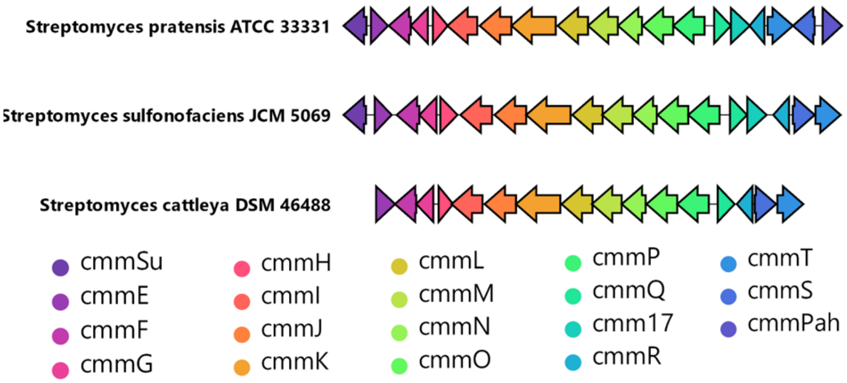

Supplementary Figure S5: Gene alignment of representative carbapenem BGCs in publicly available genomes from the NCBI database. The analysis was conducted using cblaster, a bioinformatics tool designed for comparative BGC analysis in February 2024. For comparison, the organization of the thienamycin-type BGC from *Streptomyces cattleya* is also included.

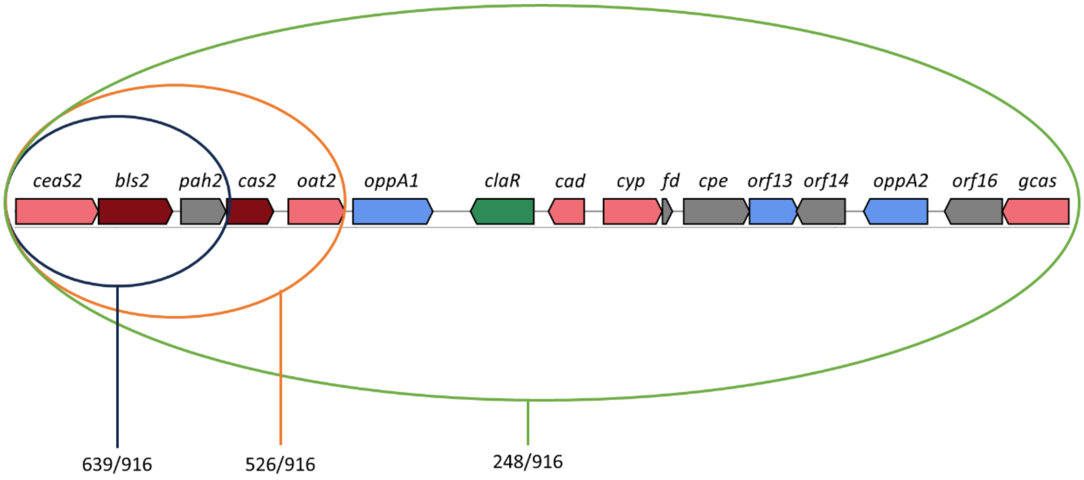

Supplementary Figure S6: Clavulanic acid-like BGCs with different gene content and coverage were analyzed based on sequences present in the publicly available NCBI database. The analysis was conducted using cblaster, a bioinformatics tool designed for comparative BGC analysis to identify homologs, in November 2024. The arrangement of genes from the CA BGC of *Streptomyces clavuligerus* is shown, with ovals representing the presence of homologous genes in the different identified instances (*n* = 916). Almost complete BGCs were identified in 248 instances, while *ceaS2-oat2* and *ceaS2-pah2* were identified in 526 and 639 instances, respectively.

Supplementary Table S1: Analysis of β-lactamase inhibitory activity production by wild type *Streptomyces pratensis* ATCC 33331 cultured on different broth and agar media. Bioactivity testing for β-lactamase inhibition was conducted using *K. pneumoniae* ATCC 15380 (with or without 60 μg/ml Penicillin G), whereas *Escherichia coli* ESS was used for detecting β-lactam antibiotic production.

Liquid media	Bioactivity detection against *K. pneumoniae*		Bioactivity detection against *E. coli* ESS	References for media[a]
	(+) PenG	(-) PenG		
Malt extract-yeast extract (MEY)	No	No	No	(1)
Mannitol-soy flour (MS)	No	No	No	(1)
R5A	No	No	No	(2)
Starch Asparagine (SA)	No	No	No	(3)
Soy fermentation media (SM)	No	No	No	(3)
Tomato Paste-Baby oatmeal (TBO)	No	No	No	(4)
Tryptic soy broth (TSB)	No	No	No	(Fisher Scientific, Canada)
**Solid media**				
**SM agar**	**Yes**	No	No	(3)
**Beef extract-starch (BES)**	**Yes**	No	No	(Fisher Scientific, Canada)
**ISP-4**	No	No	No	(Fisher Scientific, Canada)
**SA agar**	No	No	No	(3)
**Tryptic soy agar (TSA)**	No	No	No	(Fisher Scientific, Canada)

[a] References:

1. Kieser T, Bibb M, Buttner M, Chater K, Hopwood D. 2000. Practical *Streptomyces* genetics. Norwich, UK: John Innes Foundation.
2. Rodriguez M, Munez LE, Brana AF, Mendez C, Salas JA, Blanco G. 2008. Identification of transcriptional activators for thienamycin and cephamycin C biosynthetic genes within the thienamycin gene cluster from Streptomyces cattleya. Mol Microbiol 69(3) 633–645. PMID: 19138192. DOI: 10.1111/j.1365-2958.2008.06312.x
3. Paradkar, A. Jensen SE. (1995). Functional Analysis of the Gene Encoding the Clavaminate Synthase 2 Isoenzyme Involved in Clavulanic Acid Biosynthesis in *Streptomyces clavuligerus*. J Bacteriol 177(5) 1307–1314. PMID: 7868606 DOI: 10.1128/jb.177.5.1307-1314.1995
4. Higgens CE, Hamill RL, Sands TH, Hoehn MM, Davis NE, Nagarajan R, Boeck LD. 1974. The occurrence of deacetoxycephalosporin C in fungi and *Streptomycetes*. J Antibiot 27(4) 298–300. PMID: 4859396. https://doi.org/10.7164/antibiotics.27.298

Supplementary Table S2: Bacterial strains used in the current study.

Strain	Description	Resistance	Reference/source[a]
***E. coli* and other organisms**			
E. coli DH5α	General laboratory cloning host	None	Promega
E. coli ET12567/pUZ8002	DNA methylation deficient (*dam*$^-$ *dcm*$^-$ *hsdM*) conjugation host containing the plasmid pUZ8002	Chloramphenicol<br>Kanamycin	(1, 2)
E. coli BW25113/pIJ790	Host for redirect PCR targeting system	Chloramphenicol	(3)
K. pneumoniae ATCC 15380	Indicator strain for β-lactamase inhibitory bioactivity	Penicillin G	(4)
E. coli ESS	β-lactam supersensitive indicator strain	None	(5)
***Streptomyces clavuligerus* strains**			
S. clavuligerus ATCC 27064	Wild type	None	ATCC
Δ*bls*1/2	*bls*1 and *bls*2 genes deletion mutant; no production of clavulanic acid	Apramycin<br>Thiostrepton	(6, 7)
Δ*cas1/2*	cas1 and cas2 genes deletion mutant; no production of clavulanic acid	Apramycin<br>Kanamycin	(8, 6)
Δ*pah1/2*	pah1 and pah2 genes deletion mutant; no production of clavulanic acid	Apramycin<br>Thiostrepton	(9, 10)
Δ*cad*	cad gene deletion mutant; no production of clavulanic acid	Apramycin	(6)
***Streptomyces pratensis* strains**			
S. pratensis ATCC 33331	Wild type; previously known as *S. flavogriseus*	None	ATCC
Δ*ceaS-bls*	*ceaS* and *bls* genes from clavulanic acid gene cluster deletion mutant; genes replaced by apramycin cassette from pIJ773	Apramycin	This study
Δ*cmmSuEFG*	*cmmSu*, *cmmE*, *cmmF*, and *cmmG* genes from carbapenem gene cluster deletion mutant; genes replaced by apramycin cassette from pIJ773	Apramycin	This study

Δ*ceaS-bls*/Δ*cmmSuEFG*	Deletion of *cmmSu*, *cmmE*, *cmmF*, and *cmmG* (replaced by apramycin cassette from pIJ773) and deletion of *ceaS* and *bls* (replaced by hygromycin cassette from pIJ10700)	Apramycin<br>Hygromycin	This study

***Streptomyces* JAC isolates**

JAC17, 18, 19, 20, 22, 25, 26, 29, 102,128	Wild type; collected from soil samples from St. John's, Newfoundland	None	(11)
JAC18/Δ*ceaS-bls*	*ceaS* and *bls* genes deletion mutant in *Streptomyces* sp. JAC18; genes replaced by apramycin cassette from pIJ773	Apramycin	This study
JAC18/<br>Δ*cmmSuEFG*	*cmmSu*, *cmmE*, *cmmF*, and *cmmG* genes deletion mutant in *Streptomyces* sp. JAC18; genes replaced by apramycin cassette from pIJ773	Apramycin	This study
JAC25/Δ*ceaS-bls*	*ceaS* and *bls* genes deletion mutant in *Streptomyces* sp. JAC25; genes replaced by apramycin cassette from pIJ773	Apramycin	This study
JAC25/<br>Δ*cmmSuEFG*	*cmmSu*, *cmmE*, *cmmF*, and *cmmG* genes deletion mutant in *Streptomyces* sp. JAC25; genes replaced by apramycin cassette from pIJ773	Apramycin	This study
JAC128/Δ*ceaS-bls*	*ceaS* and *bls* genes deletion mutant in *Streptomyces* sp. JAC128; genes replaced by apramycin cassette from pIJ773	Apramycin	This study
JAC128/<br>Δ*cmmSuEFG*	*cmmSu*, *cmmE*, *cmmF*, and *cmmG* genes deletion mutant in *Streptomyces* sp. JAC128; genes replaced by apramycin cassette from pIJ773	Apramycin	This study
JAC18/Δ*ceaS-bls*/Δ*cmmSuEFG*	Deletion of *cmmSu*, *cmmE*, *cmmF*, and *cmmG* (replaced by apramycin cassette from pIJ773) and deletion of *ceaS* and *bls* (replaced by hygromycin cassette from pIJ10700) in *Streptomyces* sp. JAC18	Apramycin<br>Hygromycin	This study

[a] References:

1. MacNeil DJ, Gewain KM, Ruby CL, Dezeny G, Gibbons PH, MacNeil T. 1992. Analysis of *Streptomyces avermitilis* genes required for avermectin biosynthesis utilizing a novel integration vector. Gene 111(1):61-8. https://doi: 10.1016/0378-1119(92)90603-m. PMID: 1547955.
2. Paget MS, Chamberlin L, Atrih A, Foster SJ, Buttner MJ. 1999. Evidence that the extra cytoplasmic function sigma factor *sigmaE* is required for normal cell wall structure in *Streptomyces coelicolor* A3(2). J Bacteriol 181(1):204-211. https://doi:10.1128/JB.181.1.204-211.
3. Gust B, Challis GL, Fowler K, Kieser T, Chater KF. 2003. PCR-targeted *Streptomyces* gene replacement identifies a protein domain needed for biosynthesis of the sesquiterpene soil odor geosmin. Proc Natl Acad Sci U S A 100(4):1541-1546. https://doi:10.1073/pnas.0337542100
4. Reading C, Cole M. 1977. Clavulanic acid: a β-lactamase-inhibiting β-lactam from *Streptomyces clavuligerus*. Antimicrob Agents Chemother 11(5):852-857. https://doi:10.1128/AAC.11.5.852

5. Wang L, Tahlan K, Kaziuk TL, Alexander DC, Jensen SE. 2004. Transcriptional and translational analysis of the *ccaR* gene from *Streptomyces clavuligerus*. Microbiology (Reading) 150(Pt 12):4137-4145. https://doi:10.1099/mic.0.27245-0
6. Jensen SE, Elder KJ, Aidoo KA, Paradkar AS. 2000. Enzymes catalyzing the early steps of clavulanic acid biosynthesis are encoded by two sets of paralogous genes in *Streptomyces clavuligerus*. Antimicrob Agents Chemother 44(3):720-726. https://doi:10.1128/AAC.44.3.720-726.2000
7. Tahlan K, Park HU, Wong A, Beatty PH, Jensen SE. 2004. Two sets of paralogous genes encode the enzymes involved in the early stages of clavulanic acid and clavam metabolite biosynthesis in *Streptomyces clavuligerus*. Antimicrob Agents Chemother 48(3):930-939. https://doi:10.1128/AAC.48.3.930-939.2004
8. Mosher RH, Paradkar AS, Anders C, Barton B, Jensen SE. 1999. Genes specific for the biosynthesis of clavam metabolites antipodal to clavulanic acid are clustered with the gene for clavaminate synthase 1 in *Streptomyces clavuligerus*. Antimicrob Agents Chemother 43(5):1215-1224. https://doi:10.1128/AAC.43.5.1215
9. Aidoo KA, Wong A, Alexander DC, Rittammer RA, Jensen SE. 1994. Cloning, sequencing and disruption of a gene from *Streptomyces clavuligerus* involved in clavulanic acid biosynthesis. Gene 147(1):41-46. https://doi:10.1016/0378-1119(94)90036-1
10. Jensen SE, Wong A, Griffin A, Barton B. 2004. *Streptomyces clavuligerus* has a second copy of the proclavaminate amidinohydrolase gene. Antimicrob Agents Chemother 48(2):514-520. https://doi:10.1128/AAC.48.2.514-520.2004
11. Liu J, Clarke JA, McCann S, Hillier NK, Tahlan K. 2022. Analysis of Streptomyces Volatilomes Using Global Molecular Networking Reveals the Presence of Metabolites with Diverse Biological Activities. Microbiol Spectr 10(4):e0055222. https://doi:10.1128/spectrum.00552-22

Supplementary Table S3: Details of genome sequence assemblies for *Streptomyces pratensis* ATCC 33331 and the three JAC *Streptomyces* isolates, JAC18, JAC25 and JAC128 from the current study.

Attribute	*S. pratensis*	JAC18	JAC25	JAC128
Assembly length (bp)	7,413,306	7,430,941	7,484,181	7,781,095
Coverage after assembly (fold)[a]	30	52	46	48
GC content	71.17%	71.18%	71.12%	71.06%
Total number of contigs	240	176	218	370
N50	73,371	101,461	83,712	63,483
L50	32	21	27	35
Largest contig	300,279	355,458	316,823	274,800
Genome completeness (BUSCO %)[b]	98.4	98.4	98.4	97.6

[a] Indicates actual fold coverage based on estimated genome size.

[b] Calculated using the Benchmarking Universal Single-Copy Orthologs (BUSCO) software.

Supplementary Table S4: Biosynthetic gene clusters predicted in the re-sequenced genome of *S. pratensis* ATCC 33331 using antiSMASH 6.0.

Genomic Region	Chemical class	Name of similar natural product BGC	Percent similarity	MiBIG hit[a]
Region 1[b]	β-lactam	Carbapenem MM4550	58%	BGC0000842
	NRP[c] + Polyketide	SGR PTM Compound	100%	BGC0001043
	NRP	Unknown	NA[d]	NA
Region 2	NRP	Coelichelin	90%	BGC0000325
Region 3	Terpene	Isorenieratene	100%	BGC0000664
Region 4	RiPP[e]-like	Unknown	NA	NA
Region 5	β-lactam	Clavulanic acid	20%	BGC0000845
Region 6	Terpene	Hopene	69%	BGC0000663
Region 7	Polyketide	Tripartilactam/Niizalactam C	96%	BGC0002517
Region 8	RiPP-like	Unknown	NA	NA
Region 9	NRP	Cadaside A/Cadaside B	19%	BGC0001968
Region 10	Polyketide	Kinamycin	16%	BGC0000236
Region 11	Terpene	Unknown	NA	NA
Region 12	RiPP-like	Unknown	NA	NA
Region 13	Polyketide	Lactonamycin	3%	BGC0000238
Region 14	Saccharide	Istamycin	11%	BGC0000700
	Polyketide	Loseolamycin A1	32%	BGC0002362
	NRP	Rotihibin A	21%	BGC0002367
	NRP + Polyketide	Griseoviridin/Fijimiycin A	5%	BGC0000459
Region 15	Other	Desferrioxamin B/Desferrioxamine E	83%	BGC0000940
Region 16	Polyketide	Azalomycin F3a	8%	BGC0001523
Region 17	Terpene	Unknown	NA	NA
Region 18	Other	Ectoine	100%	BGC0000853
Region 19	Polyketide	Streptoketide A	100%	BGC0002081
Region 20	Polyketide: Type II polyketide + Saccharide: Hybrid/tailoring saccharide	Steffimycin D	16%	BGC0000273
Region 21	Other	Ectoine	100%	BGC0000853
	Terpene	2-methylisoborneol	100%	BGC000658
Region 22	Other	Hexacosalactone A	4%	BGC0002497
Region 23	Polyketide	Tetronasin	11%	BGC0000163
Region 24	Other	Melanin	100%	BGC0000911
Region 25	Polyketide	Spore pigment	83%	BGC0000215
	Terpene	Unknown	NA	NA
Region 26	NRP+Polyketide	Detoxin S1	100%	BGC0001840
Region 27	Butyrolactone	Unknown	NA	NA

[a] MIBiG BGC-ID: Minimal information about biosynthetic gene cluster-identification number.

[b] The top hits from regions containing sub clusters are included (27 regions and 34 BGCs).

[c] NRP: Non-ribosomal peptide.

[d] NA: Not applicable.

[e] RiPP: Ribosomally synthesized and post-translationally modified peptide.

Supplementary Figure S5: Comparison of the BGCs present in *Streptomyces pratensis* ATCC33331 and three genome sequenced *Streptomyces* isolates, JAC18, JAC25, and JAC128.

Region[a]	*S. pratensis*	JAC18	JAC25	JAC128
Region 1	Yes	Yes	Yes	Yes
Region 2	Yes	Yes	Yes	Yes
Region 3	Yes	Yes	Yes	Yes
Region 4	Yes	Yes	Yes	Yes
Region 5	Yes	Yes	Yes	Yes
Region 6	Yes	Yes	Yes	Yes
Region 7	Yes	Yes	Yes	Yes
Region 8	Yes	Yes	Yes	Yes
Region 9	Yes	Yes	Yes	Yes
Region 10	Yes	Yes	Yes	Yes
Region 11	Yes	Yes	Yes	Yes
Region 12	Yes	Yes	Yes	Yes
Region 13	Yes	Yes	Yes	Yes
Region 14	Yes	Yes	Yes	Yes
Region 15	Yes	Yes	Yes	Yes
Region 16	Yes	Yes	Yes	Yes
Region 17	Yes	Yes	Yes	Yes
Region 18	Yes	Yes	Yes	Yes
Region 19	Yes	Yes	Yes	Yes
Region 20	Yes	Yes	Yes	Yes
Region 21	Yes	Yes	Yes	Yes
Region 22	Yes	Yes	Yes	Yes
Region 23	Yes	Yes	Yes	Yes
Region 24	Yes	Yes	Yes	Yes
Region 25	Yes	Yes	Yes	Yes
Region 26	Yes	Yes	Yes	Yes
Region 27	Yes	Yes	Yes	Yes
Lanthipeptide_ JAC18 (Unknown)	No	Yes	No	Yes
NRPS_JAC128 (Unknown)	No	No	No	Yes
Lanthipeptide_JAC128 (Unknown)	No	No	No	Yes
Thioamitides_JAC128 (Unknown)	No	No	No	Yes

[a] Based on the regions predicted by antiSMASH as described in Supplementary Table S4 or those detected in the three JAC isolate genome sequences. Where indicated, entire regions were conserved between the different isolates.

Supplementary Table S6: Plasmids used in this study.

Plasmid	Description	Resistance	Reference or source[a]
pGEM®-T Easy	General cloning vector	Ampicillin	Promega
pIJ773	Template for amplification of apramycin cassette [Apr^R+oriT]	Apramycin Ampicillin	(1)
pIJ776	Template for amplification of kanamycin cassette [Kan^R+oriT]	Kanamycin Ampicillin	(2)
pIJ10700	Template for amplification of hygromycin cassette [Hyg^R+oriT]	Hygromycin Ampicillin	(3)
pCR™-Blunt II-TOPO®	Cloning vector for PCR products	Kanamycin Zeocin	Invitrogen
pGEMT-apr	pGEM®-T Easy vector containing apramycin cassette	Apramycin Ampicillin	This study
pGEMT-hyg	pGEM®-T Easy vector containing kanamycin cassette	Hygromycin Ampicillin	This study
TOPO-clav/up	pCR™-Blunt II-TOPO® vector containing upstream region of *ceaS-bls* genes from *S. pratensis*	Kanamycin Zeocin	This study
TOPO-clav/dw	pCR™-Blunt II-TOPO® vector containing downstream region of *ceaS-bls* genes from *S. pratensis*	Kanamycin Zeocin	This study
TOPO-clav/UAD	pCR™-Blunt II-TOPO® vector containing upstream and downstream region of *ceaS-bls* genes from *S. pratensis* and apramycin cassette in between the regions	Kanamycin Zeocin Apramycin	This study
TOPO-carb/up	pCR™-Blunt II-TOPO® vector containing upstream region of *cmmSuEFG* genes from *S. pratensis*	Kanamycin Zeocin	This study
pGEMT-carb-dw	pCR™-Blunt II-TOPO® vector containing upstream region of *cmmSuEFG* genes from *S. pratensis*	Ampicillin	This study
TOPO-carb/UAD	pCR™-Blunt II-TOPO® vector containing upstream and downstream region of *cmmSuEFG* genes from *S. pratensis* and apramycin cassette in between the regions	Kanamycin Zeocin Apramycin	This study
TOPO-clav/UHD	pCR™-Blunt II-TOPO® vector containing upstream and downstream region of *ceaS-bls* genes from *S. pratensis* and hygromycin cassette in between the regions	Kanamycin Zeocin Hygromycin	This study

[a] References:

1. Gust B, Challis GL, Fowler K, Kieser T, Chater KF. 2003. PCR-targeted *Streptomyces* gene replacement identifies a protein domain needed for biosynthesis of the sesquiterpene soil odor geosmin. Proc Natl Acad Sci U S A 100(4):1541-1546. https://doi:10.1073/pnas.0337542100
2. Gust B, Chandra G, Jakimowicz D, Yuqing T, Bruton CJ, Chater KF. 2004. Lambda red-mediated genetic manipulation of antibiotic-producing *Streptomyces*. Adv Appl Microbiol 54:107-128. https://doi:10.1016/S0065-2164(04)54004-2
3. Li B, Walsh CT. 2010. Identification of the gene cluster for the dithiolopyrrolone antibiotic holomycin in *Streptomyces clavuligerus*. Proc Natl Acad Sci U S A 107(46):19731-19735. https://doi:10.1073/pnas.1014140107

Supplementary Table S7: Oligonucleotide primers used in the current study.

Name	Sequence (5'→3')[a]	Purpose
Sf-*ceaS*2-F<br>Sf-*ceaS*2-R	ACGACATCTTCCCCAACGAC<br>GAGATGAAGCTGGGACCGAC	RT-PCR and confirmation of the presence of the *ceaS*2 gene in *S. pratensis* and environmental isolates
Sf-*cas2*-F<br>Sf-*cas2*-R	CGTCTACCACGACGTGTACC<br>AGGCCAGCATCACGTAGTTC	RT-PCR of *cas2* from *S. pratensis*
Sf-*orf12*-F<br>Sf-*orf12*-R	ATGATCGCGATGAGCGACAA<br>CAGCCGATCTCGAAGACCTC	RT-PCR of *orf12* (*cpe*) from *S. pratensis*
Sf-*carE*-F<br>Sf-*carE*-R	ACATCACCGACCTCTACACG<br>GTGTAACGCAGCATGAAGCC	RT-PCR and confirmation of the presence of the *cmmE* gene in *S. pratensis* and environmental isolates
Sf-*carI*-F<br>Sf-*carI*-R	TCCTGATCCGGACCATTGC<br>TACGGTGAAACTGACCGACG	RT-PCR and confirmation of the presence of the *cmmI* gene in *S. pratensis* and environmental isolates
Sf-*carM*-F<br>Sf-*carM*-R	CTGCTCACCCTGCAGATCG<br>TGGAACTCGTTGCTCCGAC	RT-PCR of *cmmM* from *S. pratensis*
Sf-*carP*-F<br>Sf-*carP*-R	CATCTGGTCCACGAGTACGG<br>CTTGTCGAGACGCATCACCT	RT-PCR and confirmation of the presence of the *cmmP* gene in *S. pratensis* and environmental isolates
Sf-*hrdB*-F<br>Sf-*hrdB*-R	CGAGTTCGGAGACCTGATCG<br>CCGTAGACCTTGCCGATCTC	RT-PCR of *hrdB* from *S. pratensis*
Sp555/6-up-F<br>Sp555/6-up-R	ATAT**GCGGCCGC**CGATGTTCCGGACTGTCA<br>GCGC**CATATG**CTTGGTCGAAACACGGGA	Forward and reverse primers to amplify the upstream region of *ceaS-bls* genes from *S. pratensis* to construct TOPO-clav/up
Sp555/6-dw2-F<br>Sp555/6-dw2-R	AT**CATATG**CTGAAGGAGACCGAGTGAAC<br>AT**GATATC**GATCGATGGTGGTCTTCACC	Forward and reverse primers to amplify the downstream region of *ceaS-bls* genes from *S. pratensis* to construct TOPO-clav/dw
car-up-F<br>*car*-up-R	ATAT**AAGCTT**GCCCTCTCGTCGAGATCATG<br>ATAT**CATATG**CTCAACTGACCACGTTCGGA	Forward and reverse primers to amplify the upstream region of *cmmSuEFG* genes from *S. pratensis* to construct TOPO-carb/up
car-dw-F<br>*car*-dw-R	ATAT**CATATG**AACCCGTTCTCGTCCAGTTC<br>ATAT**ACTAGT**CTTCGGAGTGCAGGTCAGTT	Forward and reverse primers to amplify the downstream region of *cmmSuEFG* genes from *S. pratensis* to construct TOPO-carb/dw
FRT-NdeI-F<br>FRT-NdeI-R	GCGC**CATATG**ATTCCGGGGATCCGTCGACC<br>ATAT**CATATG**TGTAGGCTGGAGCTGCTTC	Forward and reverse primers to amplify the apramycin and hygromycin cassettes to construct pGEMT-apr and pGEMT-hyg
Sp555/6-cf-F	CATGATCGCCTTCTTTCCTC	Verification of apramycin and hygromycin cassette insertion in TOPO-clav/UAD and TOPO-clav/UHD from upstream region; Verification of the *ceaS-bls* deletion region and *ceaS-bls* genes (forward)
Sp555/6-cf-R	GCAGTCGATGAGTTCGAAGG	Verification of the apramycin and hygromycin cassette insertion in TOPO-clav/UAD and TOPO-clav/UHD from downstream region
555-KO-cf-R	CGTGTCGTTGGGGAAGAT	Verification of the *ceaS-bls* genes (reverse)
Sp164-cf-F	GATCGAGACAGGCGAAGAAC	Verification of apramycin cassette insertion in TOPO-carb/UAD from upstream region
Sp164-cf-R	AATGGTCCTCACCGTGGA	Verification of apramycin cassette insertion in TOPO-carb/UAD from downstream region
cmmKO-cf-F	GCCAATCTCCTTACTGGGAAGT	Verification of the *cmmSuEFG* deletion region and *cmmSuEFG* genes (forward)

cmmKO-cf-R	TGCTGCATCTCTCCTACGAC	Verification of the *cmmSuEFG* genes (reverse)
apr-555-R	CTGTCCCTTATTCGCACCTG	Verification of the *cmmSuEFG* and *ceaS-bls* deletion region (reverse)
cmm17-cf-F<br>*cmm17*-cf-R	GCCTGCGACTTCGTACTGAT<br>GTCTCTTCACGGAGCCTGTC	Confirmation of the presence of the *cmm17* gene in *S. pratensis* and environmental isolates
car-sp-cf-F<br>*car*-sp-cf-R	CTCGACATCCTGGTCAACAA<br>CCCTGGTTCCTGATCAGTTC	Confirmation of the presence of the *car* (*cad*) gene in *S. pratensis* and environmental isolates
gcas-sp-cf-F<br>*gcas*-sp-cf-R	CCTCGTTCTCCGACTACCAC<br>CCACCAGTACATGAGGGATG	Confirmation of the presence of the *gcas* gene in *S. pratensis* and environmental isolates
ccaR-sp-cf-F<br>*ccaR*-sp-cf-R	CTCCATGATTTCCCAGAACG<br>TCATCCACACACTCGTCTGC	Confirmation of the presence of the *ccaR* gene in *S. pratensis* and environmental isolates
16s-27F<br>16s-1492R	AGAGTTTGATCCTGGCTCAG<br>ACGGCTACCTTGTTACGACT	Amplification of 16s rRNA gene and its sequencing

[a] Non-homologous extensions are underlined, and restriction sites are indicated in bold.
